# Single‐Cell and Spatial Transcriptomics Decodes Wharton's Jelly‐Derived Mesenchymal Stem Cells Heterogeneity and a Subpopulation with Wound Repair Signatures

**DOI:** 10.1002/advs.202204786

**Published:** 2022-12-11

**Authors:** Penghong Chen, Shijie Tang, Ming Li, Dezhi Wang, Caixiang Chen, Yiqun Qiu, Zhuoqun Fang, Haoruo Zhang, Hangqi Gao, Haiyan Weng, Kailun Hu, Jian Lin, Qingxia Lin, Yi Tan, Shirong Li, Jinghua Chen, Liangwan Chen, Xiaosong Chen

**Affiliations:** ^1^ Department of Plastic Surgery and Regenerative Medicine Fujian Medical University Union Hospital Fuzhou 350001 China; ^2^ Department of Plastic Surgery and Regenerative Medicine Institute Fujian Medical University Fuzhou 350001 China; ^3^ Engineering Research Center of Tissue and Organ Regeneration Fujian Province University Fuzhou 350001 China; ^4^ Department of Stem Cell Research Institute Fujian Medical University Fuzhou 350004 China; ^5^ Department of Obstetrics Quanzhou Women and Children's Hospital Quanzhou 362000 China; ^6^ Qilu Cell Therapy Technology Co., Ltd Jinan Shandong 250000 China; ^7^ Department of Plastic and Reconstructive Surgery Shinrong Plastic Surgery Hospital Chongqing 401120 China; ^8^ Department of Pharmaceutical Analysis, the School of Pharmacy Fujian Medical University Fuzhou 350100 China; ^9^ Department of Cardiac Surgery Fujian Medical University Union Hospital Fuzhou 350001 China

**Keywords:** heterogeneity, single‐cell RNA sequencing, spatial transcriptome, subpopulations, Wharton's jelly‐derived mesenchymal stem cells

## Abstract

The highly heterogeneous characteristics of Wharton's jelly mesenchymal stem cells (WJ‐MSCs) may be responsible for the poor clinical outcomes and poor reproducibility of treatments based on WJ‐MSCs. Exploration of WJ‐MSC heterogeneity with multimodal single‐cell technologies will aid in establishing accurate MSC subtyping and developing screening protocols for dominant functional subpopulations. Here, the characteristics of WJ‐MSCs are systematically analyzed by single cell and spatial transcriptome sequencing. Single‐cell transcriptomics analysis identifies four WJ‐MSC subpopulations, namely proliferative_MSCs, niche‐supporting_MSCs, metabolism‐related_MSCs and biofunctional‐type_MSCs. Furthermore, the transcriptome, cellular heterogeneity, and cell‐state trajectories of these subpopulations are characterized. Intriguingly, the biofunctional‐type MSCs (marked by S100A9, CD29, and CD142) selected in this study exhibit promising wound repair properties in vitro and in vivo. Finally, by integrating omics data, it has been found that the S100A9^+^CD29^+^CD142^+^ subpopulation is more enriched in the fetal segment of the umbilical cord, suggesting that this subpopulation deriving from the fetal segment may have potential for developing into an ideal therapeutic agent for wound healing. Overall, the presented study comprehensively maps the heterogeneity of WJ‐MSCs and provides an essential resource for future development of WJ‐MSC‐based drugs.

## Graphical abstract

The heterogeneity of Wharton's jelly mesenchymal stem cells (WJ‐MSCs) hampers clinical translation. The authors map a comprehensive single‐cell and spatial transcriptomic atlas to decipher the heterogeneity of WJ‐MSCs, and verify the efficient wound repair capabilities of the S100A9^+^CD29^+^CD142^+^ subpopulations, which are more enriched in the fetal segment of an umbilical cord, providing a more targeted way to treat diseases.

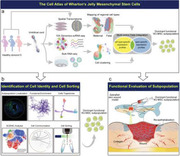

## 1 Introduction

Mesenchymal stem cells (MSCs) are multipotent cells with the ability to self‐renew and are being studied extensively as a cell therapy.^[^ ^1^ ^]^ Wharton's jelly MSCs (WJ‐MSCs) have gained widespread favor over the last few years owing to their inherent advantages over MSCs from other sources, such as faster proliferation, greater ex vivo expansion capabilities,^[^ ^2^ ^]^ a lower incidence of graft versus host disease and lack of risk of teratomas.^[^ ^3^ ^]^ To date, hundreds of clinical trials have been registered on Clinical Trial.gov using WJ‐MSCs to treat a multitude of diseases. Despite the encouraging efficacy of such therapeutic modalities, most of the trials failed to replicate a significant improvement.^[^ ^4^ ^]^ The heterogeneity and disparity of MSCs has been considered an obstacle for clinical translation into reproducible, predictable, and standardized therapeutic approaches.^[^ ^5^ ^]^ Recent studies have shown that the variability of gene expression profiles of living MSCs used for therapies was derived from interindividual heterogeneity (e.g., heterogeneity in age, sex, and phenotype),^[^ ^5^, ^6^ ^]^ tissue‐dependent heterogeneity,^[^ ^7^ ^]^ and the use of different isolation methods,^[^ ^8^ ^]^ passages^[^ ^9^ ^]^ and subsets of MSCs.^[^ ^10^ ^]^ Obviously, the differences above lead to poor homogeneity of MSC products, calling into question the degree to which such products can achieve a clinical benefit. The standardization of MSC populations and the identification of specific subpopulations of MSCs should thus be a top research priority to facilitate their effective translation to clinical usage. However, clear insights into the identity, biology, and function of these cells are still lacking.

Single‐cell RNA sequencing (scRNA‐seq) allows detailed characterization of MSCs at unprecedented molecular resolution. Although scholars have recently defined the transcriptomic features of WJ‐MSCs, the number of investigated cells was insufficient, and the function of each cell subset lacked an unambiguous definition.^[^ ^11^ ^]^ A common limitation of scRNA‐seq studies is that crucial spatial information of WJ‐MSCs is often lost after cellular dissociation, thus limiting our understanding of cell‐to‐cell interactions and functional organization in situ. Building on recent advances, the spatial transcriptomics (ST) method has overcome this limitation.^[^ ^12^ ^]^ Notably, the spatially heterogeneous distribution of these cells leads to functional variations among cells located in different umbilical cord (UC) regions,^[^ ^13^ ^]^ which indicates that the potential importance of WJ‐MSC spatial heterogeneity for clinical treatment. However, a full characterization of MSCs from different parts of the WJ by ST remains to be reported.

**Figure 1:**
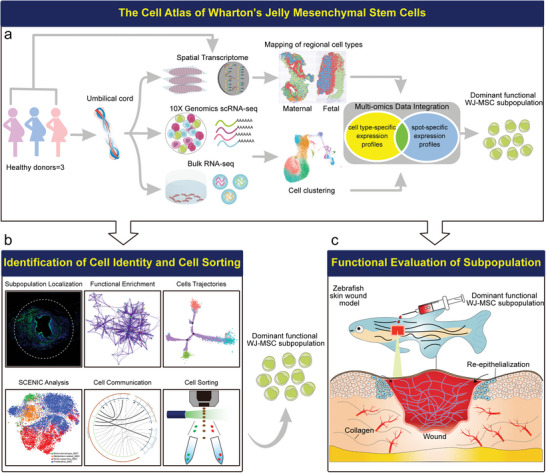
Scheme. a) Single‐cell transcriptome combined with spatial transcriptome mapping of spatiotemporal heterogeneity of WJ‐MSCs. b) Further bioinformatics analysis of WJ‐MSCs subpopulations. c) Functional verification of dominant functional subpopulation.

In this context, we aimed to construct a comprehensive and longitudinal view of spatiotemporal heterogeneity mapping of WJ‐MSCs and, more importantly, to decipher which MSC subpopulation on which area would be the optimal seed cells for repairing injured tissue upon transplantation (**Figure** **1**a–c). Here, the transcriptomes of 38 125 single‐cells from culture‐expanded WJ‐MSCs were analyzed by scRNA‐seq at passage 0 (P0) and passage 3 (P3). We reported four subpopulations of WJ‐MSCs with different molecular signatures that could potentially reflect their respective functional characteristics. Further analysis predicted candidates’ transcription factors (TFs) that may modulate cell fate and revealed interactive relationships among subpopulations, with a particular focus on a S100A9^+^CD29^+^CD142^+^ subpopulation that exhibited enhanced tissue restoration capabilities in a zebrafish wound healing model. Then, ST was performed to draw an unbiased map of the expressed transcripts in maternal and fetal segments of the same UC. Finally, we integrated single‐cell and spatial transcriptomic data to determine the spatial distribution of the different cell types, and the fetal part of the UC was identified as the potential and ideal cellular and spatial sources for wound repair.

## 2 Results

### 2.1 Single‐Cell Transcriptome Atlas of WJ‐MSC

**Figure 2:**
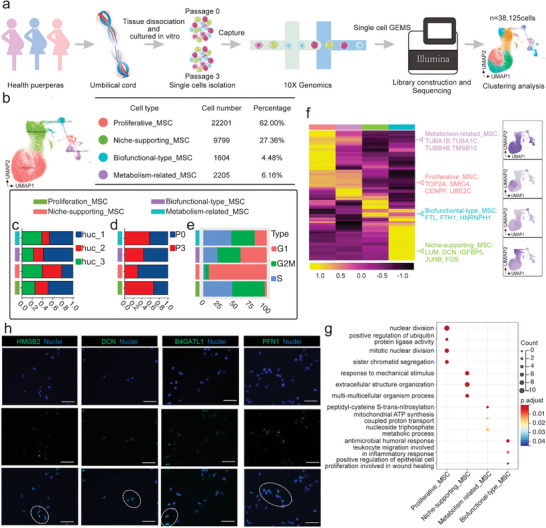
Identification of WJ‐MSC subpopulations. a) Diagram of experimental design and downstream bioinformatics analysis of scRNA‐seq. b) UMAP visualization of four different WJ‐MSC subpopulations. Colors represent subpopulations. c) Proportions of different WJ‐MSC subpopulations among different donors. d) Proportions of different WJ‐MSC subpopulations between passages. e) Distribution of different WJ‐MSC subpopulations across the cell cycle. f) Gene expression heatmap of four different WJ‐MSC subpopulations. g) GO biological processes (GO‐BP) enrichment analysis of four different WJ‐MSC subpopulations. h) Immunostaining of B4GATL1, DCN, HMGB2, and PFN1 in WJ‐MSCs. Scale bar: 120 µm.

To understand the cellular diversity and transcriptional signatures of WJ‐MSCs from different generations, we first isolated WJ‐MSCs from 3 human UCs, expanded them in vitro, and then collected single cells from P0 and P3 generation for subsequent 10× Genomics Chromium scRNA‐seq (**Figure** **2**a; Figure S1a and Table S1, Supporting Information). These in vitro expanded WJ‐MSCs met the requirements of MSCs definition. A total of 38 125 single cells from 6 samples (named as huc_1P0, huc_1P3, huc_2P0, huc_2P3, huc_3P0, and huc_3P3, respectively) were subjected to downstream analyses after filtering with Seurat, and the expression of a median of 4879 genes per cell and a median of 28 774 unique molecular identifiers (UMIs) counts per cell could be detected (Figure S1b,c, Supporting Information), suggesting that our data were of high quality. We further investigated the expression of classic MSC genes according to the MSC definition,^[^ ^14^ ^]^ such as cell surface positivity (UMI > 0) for CD73, CD90 and CD105, and negativity (UMI = 0) for CD31, CD34, CD45 and HLA‐DR (Figure S2a–c, Supporting Information). The flow cytometry results also verified the MSC phenotype (Figure S3, Supporting Information). Next, unsupervised graph clustering partitioned the retained cells into 13 clusters (Figure S4a–c and Table S2, Supporting Information), which were categorized into cell types based on marker gene expression (Figure S4d, Supporting Information). Compared with other cell clusters, Cluster 0, 2, 3 and 7 highly expressed well‐known proliferation‐related genes, such as UBE2C, TOP2A, HMGB2, CDC20, PCNA and HIF1A,^[^ ^15^ ^]^ implying that these subpopulations possess a higher proliferative capacity. The results of Gene Ontology (GO) enrichment analysis demonstrated that these three subsets were also significantly enriched in nuclear division, DNA replication, and stem cell proliferation (Figure S5a, Supporting Information). In contrast to the other subpopulations, clusters 1, 4 and 11 were marked by ACTA2, TGM2, FOS, TGFB1, FLNA and COL3A1. Biological processes such as response to corticosteroids, pericyte cell differentiation, putrescine catabolic process, and extracellular matrix (ECM) organization were enriched in this group. Cluster 10 was marked by S100A10, PDLIM1 and CAV1; clusters 5, 8 and 12 were characterized by the expression of UBE2S, TAGLN, TPM1 and TMSB4X; and clusters 6 and 9 were marked by B4GALT1, HEG1, CXCL3, CXCL1 and HMOX1, indicating their regenerative and immunomodulatory therapeutic potential. The gene expression profiles of different WJ‐MSC subpopulations reflected different molecular signatures. In addition, individual subpopulations were annotated using SingleR (Figure S5b, Supporting Information). We eventually validated the identity of these candidate subpopulations based on expression profiles, functional similarities, and the literature (Table S3, Supporting Information). The relevant subpopulations were pooled for analysis and named the “proliferative_MSC” (clusters 0, 2, 3, and 7), “niche‐supporting_MSC” (clusters 1, 4, and 11), “metabolism‐related_MSC” (clusters 5, 8, and 12), and “biofunctional‐type_MSC” (clusters 6 and 9) subpopulations (Figure 2b,f,g; Figure S5c, Supporting Information). Cluster 10 accounted for a small proportion of cells and lacked functional similarity with other clusters, so this cluster was removed from the subsequent analysis. Overall, these cell types can be defined by multiple genes. Moreover, the pairwise subpopulation correlation analysis indicated close relationships between the metabolism‐related_MSC and proliferative_MSC, proliferative_MSC and biofunctional‐type_MSC, and biofunctional‐type_MSC and niche‐supporting_MSC subpopulations (Figure S5d, Supporting Information). We also found that cells from different donors and different passages have heterogeneous proportions of different cell types (Figure 2c,d). Interestingly, the proliferative_MSC subpopulation accounted for the largest proportion of cells among all populations, and most of the cells were assigned to the G2/M and S cell cycle phases, which reflected the self‐renewal ability of in vitro‐cultured WJ‐MSCs and the unique properties of MSCs (Figure 2b,e). Moreover, the proportion of P3 proliferative_MSCs in G2/M and S phases was larger than that of P0 proliferative_MSCs (Figure S5e, Supporting Information), implying that the proliferation of cultured MSCs increased with the culture time at early passages. Immunofluorescence assays corroborated the presence of four subpopulations (Figure 2h) and suggested that the subpopulation analyses effectively defined unique gene expression patterns in WJ‐MSC subgroups that are likely obscured in bulk RNA‐seq analyses.

### 2.2 Dynamic Transcriptional Changes and Distinct Transcriptional Controls in WJ‐MSC Subpopulations

**Figure 3:**
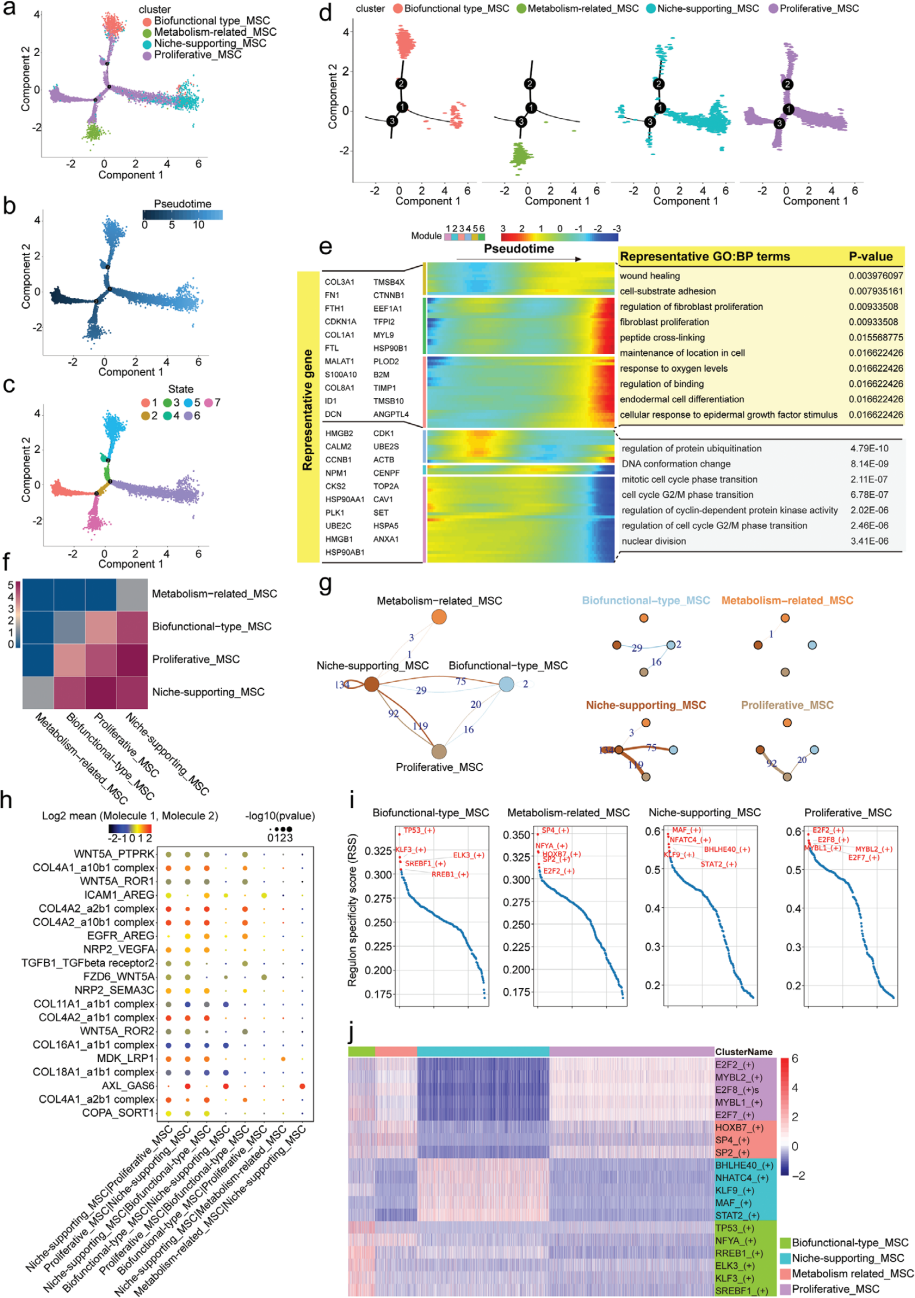
Pseudotime analysis, cell–cell communication, and TF analysis for WJ‐MSC subpopulations. a) Distribution by cluster trajectory. b) Pseudotemporal ordering trajectory map. Dark to light colors represent the pseudotime order. c) Pseudotime reflecting the cell state transition. d) Track of differentiation of the four subgroups. e) The trajectory map of differential gene pseudotemporal expression and GO enrichment of modules. f) Heatmap of protein interaction numbers among four WJ‐MSC subpopulations. The value is a log conversion of the number of interactions (*p* < 0.05). g) The interaction network of WJ‐MSC subpopulations. The numbers represent the numbers of ligand–receptor pairs (*p* < 0.05). h) Bubble plot showing subpopulation interaction pairing. Larger dots indicate smaller *p* values. i) The top 5 specific regulators of each WJ‐MSC subpopulation. j) Heatmap showing the top 5 regulators of each WJ‐MSC subpopulation.

We further depicted the potential differentiation process of the identified WJ‐MSC populations by using the R package monocle and constructed cell state trajectories. We performed a pseudotime analysis of the P0 and P3 samples, respectively. The results showed that the trajectories of four subpopulations at passage P0 could not be well separated (Figure S6a–d, Supporting Information). The trajectories of each subpopulation were relatively similar, most probably because of the subpopulations in primary WJ‐MSCs has a similar transcriptional status, and cell transitions were not sufficient under the current transcriptional state. Subsequently, we checked the distribution of subpopulations at passage P3 (**Figure** **3**a–c). These cells were chronologically split into 7 cellular states based on pseudotime ordering, in which the starting point was state 1 and the stopping point was state 7 (Figure 3c), which indicated that the cells developed in a strict time order. Proliferative_MSCs were the starting point of the trajectory, and exited throughout the entire trajectory, while biofunctional‐type_MSCs, niche‐supporting_MSCs and metabolism‐related_MSCs were mainly distributed in the end (Figure 3a,d). Namely, some proliferative_MSCs underwent serial mitotic proliferation, while others completed the transition between cell subpopulations.

We next examined the dynamic gene expression profiles of these four subclusters and assigned them into six modules according to pseudotemporal expression patterns (Figure 3e). Along the trajectory following the pseudotime, all the dynamically expressed genes showed two main trends. These included genes gradually upregulated (Module 2, 4, and 6) and genes gradually downregulated (Module 1, 3, and 5). We noticed that the representative expressed genes (COL3A1, FN1, FTH1, COL1A1, DCN, and MYL9) with an uptrend were enriched in wound healing, cell‐substrate adhesion, regulation of fibroblast proliferation and response to oxygen levels, while the decreasing genes were involved in processes including regulation of protein ubiquitination, mitotic cell cycle phase transition, and nuclear division. This change reflected the process of WJ‐MSC subpopulations evolution or the change from proliferative_MSCs to other three subpopulations (niche‐supporting_MSCs, biofunctional‐type_MSCs, and metabolism‐related_MSCs), consistent with the results of the pseudotime analysis using four WJ‐MSC subpopulations (Figure 3a). Taken together, these data further supported that WJ‐MSCs are transcriptionally and developmentally heterogeneous, and revealed close relationships among WJ‐MSCs subpopulations.

### 2.3 Cell–Cell Communication Networks

To further characterize the crosstalk between WJ‐MSC subsets, CellPhoneDB (www.cellphonedb.org) was utilized to infer the unbiased receptor–ligand interaction among these cell clusters.^[^ ^16^ ^]^ This analysis revealed that niche‐supporting_MSCs are the most active ligand senders, suggesting that these cells may be fundamental for maintaining the fine homeostasis and functional specificity of cell subsets (Figure 3f,g). Notably, we found that niche‐supporting_MSCs expressed relatively high levels of COL4A1, COL4A2, COL18A1, ICAM1, WNT5A and MDK, while the corresponding partners of these factors, such as the a10b1 complex, ROR1, AREG, VEGFA and LRP1, were widely expressed in proliferative_MSCs, metabolism‐related_MSCs and biofunctional‐type_MSCs (Figure 3h). These ligand–receptor pairs may be related to ECM reconstruction, tissue development, intracellular signaling, angiogenesis, and cell proliferation. Overall, while they are clearly preliminary, our data indicate that the complex crosstalk that occurs among these clusters via diverse ligand–receptor signaling pathways could have a substantial impact on normal biological processes during in vitro culture.

### 2.4 Mapping Specific Regulon Networks via SCENIC

The divergent gene expression repertoires of different MSC subgroups suggested that gene expression is governed by diverse upstream regulatory mechanisms. However, the exact mechanism of how WJ‐MSC subgroup fate choices are regulated during in vitro expansion remains elusive. Here, single‐cell regulatory network inference and clustering (SCENIC) pipeline was applied to further reconstruct gene‐regulatory networks and infer the differences in the potential regulon activity of cellular clusters.^[^ ^17^ ^]^ The unsupervised clusters constructed by using these TF regulatory network signatures were different from the clusters constructed by using gene expression alone (Figure S7a, Supporting Information). Because gene transcription is orchestrated by multiple TFs (Table S4, Supporting Information), we calculated the regulon‐specific scores of clusters and ranked the top 5 identified TFs based on the Jensen–Shannon (JS) divergence algorithm (Figure 3i; Table S5, Supporting Information).

Additionally, the identity of WJ‐MSC subtypes can be distinguished based on the expression patterns of the representative TFs in four clusters (Figure 3j); for example, higher activity of TP53 and ELK3 was observed in biofunctional‐type_MSCs, upregulation of SP4, NFYA, HOXB7 and SP2 was observed in metabolism‐related_MSCs, elevated activity of MAF, NFATC4 and KLF9 was observed in niche‐supporting_MSCs, and E2F2, E2F8 and MYBL1 expression was observed in proliferative_MSCs. We also predicted putative target genes of the top 5 TFs and validated their gene functions via the miRNet database (Figure S7b, Supporting Information). Collectively, these data indicate that the combinations of multiple TFs regulate WJ‐MSC development and control cellular functions to maintain the heterogeneous states, which deepen our understanding of the functional heterogeneity of WJ‐MSCs and the underlying regulatory basis.

### 2.5 Distinct Transcriptional Signatures of WJ‐MSC Subtypes at P3 and P0

**Figure 4:**
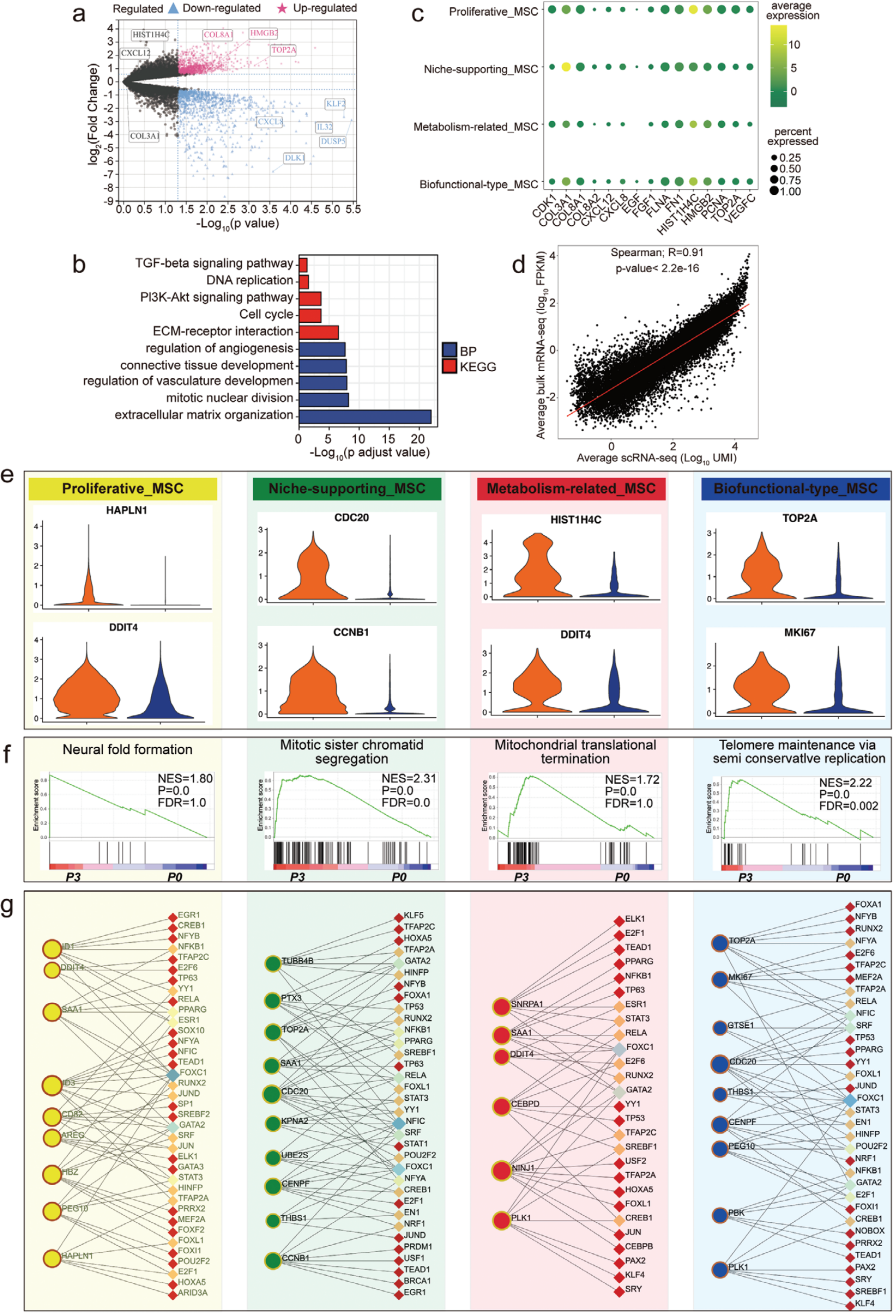
Transcriptional characteristics and regulatory mechanisms of P3 and P0 WJ‐MSC subpopulations. a) Volcano plot of differential gene expression between P3 and P0. b) The functional enrichment analysis of DEGs of P3. c) Bubble plot showing the relative expression changes of specific genes in different cell types. The larger the circle is, the greater the percentage of expression, and the color from yellow to green indicates decreasing average expression. d) Correlation analysis of bulk RNA‐seq and scRNA‐seq data. e) Gene expression differences in four WJ‐MSC subpopulations between P3 and P0. f) The functional enrichment analysis of DEGs of the four WJ‐MSC subpopulations (P3 versus P0). g) TFs prediction for the top 10 DEGs of the four WJ‐MSC subpopulations (P3 versus P0). Subnetworks with at least 3 nodes are listed below.

To define gene expression changes in global transcriptome patterns and validate the completeness of the scRNA‐seq data, we also performed bulk RNA‐seq analysis of P3 (*n* = 3) and P0 (*n* = 3) MSC samples in parallel. The bulk RNA‐seq results were reasonable (Figure S8a,b, Supporting Information), and all samples expressed MSC markers (Figure S8c, Supporting Information). A total of 532 upregulated genes and 695 downregulated genes were screened in P3 versus P0 WJ‐MSCs (**Figure** **4**a; Table S6, Supporting Information). Subsequently, functional enrichment analyses indicated that DNA replication, the cell cycle, regulation of angiogenesis, and ECM organization were specifically activated in the P3 group (Figure 4b). Overall, these data show that the significant heterogeneity of WJ‐MSCs at different passages was derived from characteristic gene expression profiles.

We next explored the expression levels of differential genes (bulk) in different MSC subpopulations (single cell) (Figure 4c; Table S7, Supporting Information). TOP2A is an upregulated gene in bulk RNA‐seq and is wide‐spread with high expression level in all subpopulations, while HIST1H4C is shown to be unchanged, mainly in proliferative_MSC. Conversely, CXCL8 gene expression was downregulated in bulk RNA‐seq and highly expressed in niche‐supporting_MSC. These findings reflect that cellular diversity and molecular complexity of WJ‐MSCs, further emphasizing the advantages of single cell analysis over studies of population averages. Importantly, we observed a significant positive trend between the bulk and scRNA‐seq data with a Spearman correlation of 0.91 (Figure 4d), and thus excluding biases during the single‐cell dissociation.

Previous studies suggest that the passage number is also reported to affect MSCs potency.^[^ ^18^ ^]^ Thus, we next explored the cellular heterogeneity between generations and their pertinent regulatory mechanisms. We found that WJ‐MSCs subpopulations differ in propensity for differentiation into different lineages according to lineages differentiation score^[^ ^19^ ^]^ (Figure S8d–g, Supporting Information), as we know the heterogeneity in lineage commitments of hematopoietic stem cells subpopulations.^[^ ^20^ ^]^ As shown in the Figure S8d,e, Supporting Information, niche‐supporting_MSC have a stronger chondrogenic and osteogenic potential than other MSC subpopulations, which is associated with increased differentiation‐related genes. Such intrinsic differences in differentiation capacity could benefit personalized clinical therapy. The transcriptional analysis revealed the unique patterns of genes of four cell types between P3 and P0 (Figure 4e). Then, gene set enrichment analysis (GSEA) was used to globally investigate the differences, the results revealed that proliferative_MSC possess gene expression related to neural fold formation, niche‐supporting_MSC preferentially engaged mitotic sister chromatid segregation, metabolism‐related_MSC were enriched in mitochondrial translational termination, and biofunctional‐type_MSC were enriched in telomere maintenance via semi conservative replication (Figure 4f). In addition, we asked whether the differentially expressed genes (DEGs) between P3 and P0 in each subpopulation are also under the unique transcriptional controls and achieved transcriptional activation by the activities of TF combinations. Interconnected TF‐gene network revealed that the majority of the DEGs are regulated by the combination of multiple TFs (Figure 4g). These results demonstrate that transcriptionally defined WJ‐MSCs subpopulations possessed unique cellular and functional properties and are maintained by distinct transcriptional networks.

### 2.6 Heterogeneous Proliferative_MSC Subpopulations Cooperate to Regulate Cell Proliferation Activities

**Figure 5:**
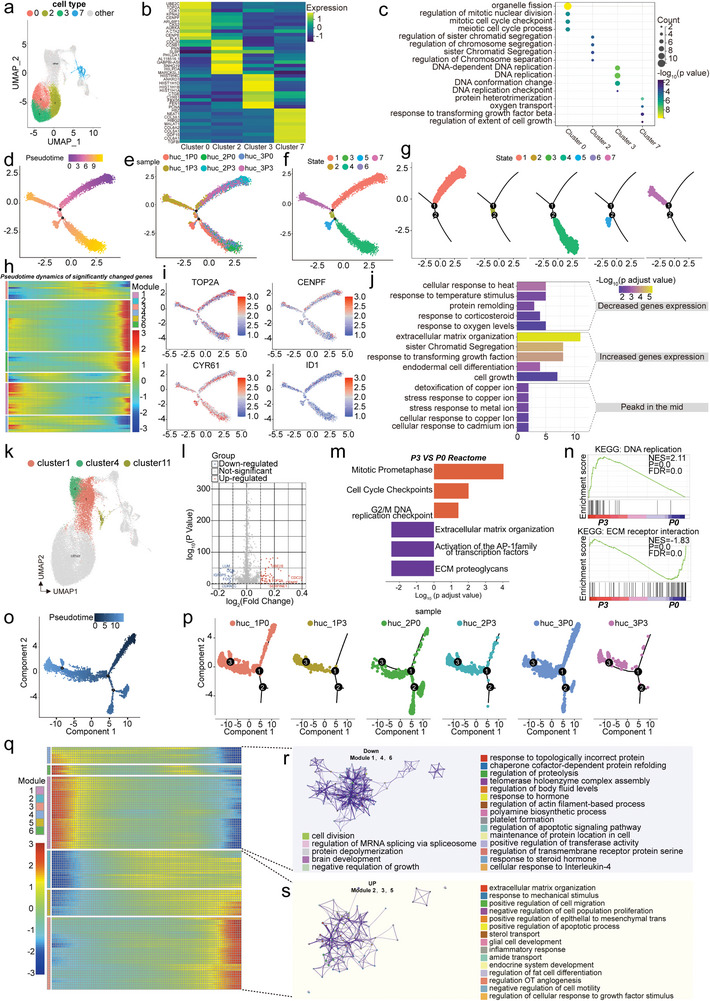
Heterogeneity analysis of proliferative_MSCs and niche‐supporting_MSCs. a) UMAP visualization of four proliferative_MSC subsets. b) Heatmap shows DEGs of proliferative_MSC subsets. c) GO analysis of proliferative_MSC subsets. d) Pseudotime analysis of proliferative_MSC subsets. e) Trajectory analysis by sample source. f) Trajectory analysis by state. g) Cell trajectory diagram for each state. h) Clustering heatmap of DEGs over pseudotime. i) Expression trend of TOP2A, CENPF, CYR61, and ID1 along the trajectory. j) GO enrichment analysis of gene modules. k) UMAP visualization the distributions of niche‐supporting_MSCs subsets. l) DEGs between P3 niche‐supporting_MSCs and P0 niche‐supporting_MSCs. The upregulated genes were identified by logFC > 0.1 and *p* value < 0.05. m) Reactome analysis of DEG functional enrichment. Red indicates an upregulated gene enrichment pathway, and blue indicates a downregulated gene enrichment pathway. n) GSEA‐KEGG showing the upward and downward trend for the P3 niche‐supporting_MSC gene set, respectively. o) Pseudotime trajectory for dimension reduction. p) Split the trajectory analysis chart by sample to show the trajectory distribution of samples in different passages. q) Heatmap of the top 100 significantly changed genes in 6 gene modules. r) GO‐BP enrichment analysis of gene modules with down‐regulated trends. s) GO‐BP enrichment analysis of gene modules with upward trend.

The proliferative_MSCs is the largest proportion of WJ‐MSCs, and we identified other specific functions in addition to their proliferative potential. The results showed that top genes were enriched in pathways related to female reproductive development, immune regulation and nitric oxide biosynthetic process (Figure S9a,b, Supporting Information), implying their role as promising therapeutic agents in female reproductive system. We next explored the heterogeneity of proliferative_MSCs. Proliferative_MSCs were represented by clusters 0, 2, 3, and 7 (**Figure** **5**a). The distribution of four clusters from different donors and different passages is shown (Figure S9c,d, Supporting Information). These clusters were isolated to compare genes in their signatures. The expression profiles of distinct subclusters of cells are presented (Figure 5b; Figure S9e,f, Supporting Information). Characteristic gene signatures were identified in each cluster, for example, UBE2C, TOP2A and MKI67 in cluster 0; CDC20 and CENPF in cluster 2; HIST1H4C, HIST1H1D, HIST1H1B and HIST1H1A in cluster 3; and NEAT1, COL3A1 and MALAT1 in cluster 7 (Figure S9f, Supporting Information). GO analysis of DEGs in the four subpopulations revealed that cell cycle processes were enriched in cluster 0, the regulation of chromosome segregation was enriched in cluster 2, DNA replication was significantly enriched in cluster 3, and protein heterotrimerization, oxygen transport and response to transforming growth factor beta were enriched in cluster 7 (Figure 5f). Concurrently, there was also an overlap in the function of genes (Figure S9g, Supporting Information). These findings show that proliferative_MSCs are highly heterogeneous and composed of multiple functionally distinct cell subsets.

Subsequently, the developmental trajectory was explored through pseudotime analysis. We ordered the cells in pseudotime and arranged most of the proliferative_MSCs into a major trajectory (Figure 5d,e). Along the trajectory progression, the cells experienced two bifurcations and 5 states (Figure 5f). According to sample distribution trends along the trajectories, P3 cells were mainly located in state 1 (Figure 5g). Of note, is that the expression of proliferation marker genes (CENPF, H2AFX, HMGB1, MKI67, PCNA, and TOP2A) was higher in state 1 than in other states (Figure S9h, Supporting Information), demonstrating that the proliferative capacity of P3 cells is high. Then, significantly affected genes along the trajectory following the pseudotime were assigned to 6 modules (Figure 5h,i). Functional enrichment analysis of these dynamically expressed genes indicated that cellular response to heat, response to temperature stimulus, and response to oxygen levels were markedly dysregulated (Figure 5j). The genes showed an up‐regulated trend in modules 2, 5 and 6, several important biological processes for WJ‐MSC development, including cell growth, ECM organization and response to transforming growth factor beta, were enriched (Figure 5j). In addition, gene expression activities of ACTB, ID1, AREG and UBE2C from module 3 were upregulated along corresponding pseudotime, reaching a peak near the midpoint of the trajectory, and they were associated with detoxification of copper ions, stress response to copper ions and cellular response to cadmium ions (Figure 5j). Overall, the dynamic gene expression patterns reflect their roles in cell growth and development.

### 2.7 Three Different Niche‐Supporting_MSC Subtypes Were Identified

Niche‐supporting_MSCs were clustered into three different types (Figure 5k; Figure S10a–c, Supporting Information). Each subcluster can be defined by its own distinctive gene signatures (Figure S10d,f, Supporting Information). GO analysis showed that the regulation of endocytosis and aging were enriched in cluster 1 (Figure S10e, Supporting Information), suggesting that cluster 1 may mediate intercellular communication. In cluster 4, the expression of DCN, COL3A1, COL14A1 and LUM was enriched (Figure S10f, Supporting Information); these factors are associated with collagen fibril organization, ECM organization and tissue remodeling (Figure S10e, Supporting Information), implying that this cluster provides structural support for cell development. Cluster 11 exhibited enrichment of genes involved in reproductive structure development and positive regulation of reactive oxygen species metabolic processes (Figure S10e, Supporting Information).

We next explored the potential differences between niche‐supporting_MSCs at P3 and P0 by examining significant DEGs. Several proliferation‐associated genes, such as CENPF, UBE2S and TOP2A, showed higher expression levels in P3 cells than in P0 cells, while the expression of DCN, FOS and LUM was relatively downregulated (Figure 5l). Reactome analyses of these DEGs indicated that the upregulated genes were enriched in cell cycle and mitotic prometaphase, and the downregulated genes were involved in ECM organization (Figure 5m). These findings are consistent with the gene set enrichment analysis (GSEA) results, which revealed enrichment of Kyoto Encylopedia of Genes and Genomes (KEGG) DNA replication and KEGG ECM receptor interaction (Figure 5n) in P3 cells and P0 cells, respectively. Thus, we propose that niche‐supporting_MSCs from P0 may partly inherit the collagen‐rich property of WJ tissues, while niche‐supporting_MSC from P3 meet high proliferation signatures of cultured‐MSCs.

We further constructed a transcriptional trajectory of these cells (Figure 5o; Figure S10g, Supporting Information). The results clearly demonstrated the distribution of P0 cells were distributed along each trajectory. In contrast, P3 cells were concentrated near the left side of the trajectory (Figure 5p), perhaps reflecting different stages at which the niche‐supporting_MSC subpopulation underwent a transformation during in vitro expansion. We also identified several distinct patterns of gene expression after assigning pseudotime values to the cells (Figure 5q; Table S8, Supporting Information). This analysis revealed six gene modules, which can be divided into significantly upregulated (module 1, module 4, and module 6) and downregulated (module 2, module 3, and module 5) with pseudotime. Gene ontology analysis of the downregulated modules revealed enrichment of response to topologically incorrect proteins, regulation of proteolysis, and regulation of actin filament‐based processes (Figure 5r; Table S9, Supporting Information). The upregulated modules were enriched in genes associated with response to mechanical stimulus, positive regulation of cell migration, regulation of angiogenesis, and regulation of cellular response to growth factor stimulation (Figure 5s; Table S9, Supporting Information). Collectively, our work revealed the distinct cellular processes and functional shifts in niche‐supporting_MSC subpopulations derived from different passages.

### 2.8 Biofunctional‐Type_MSC Subclusters Orchestration of the Wound Healing Response

**Figure 6:**
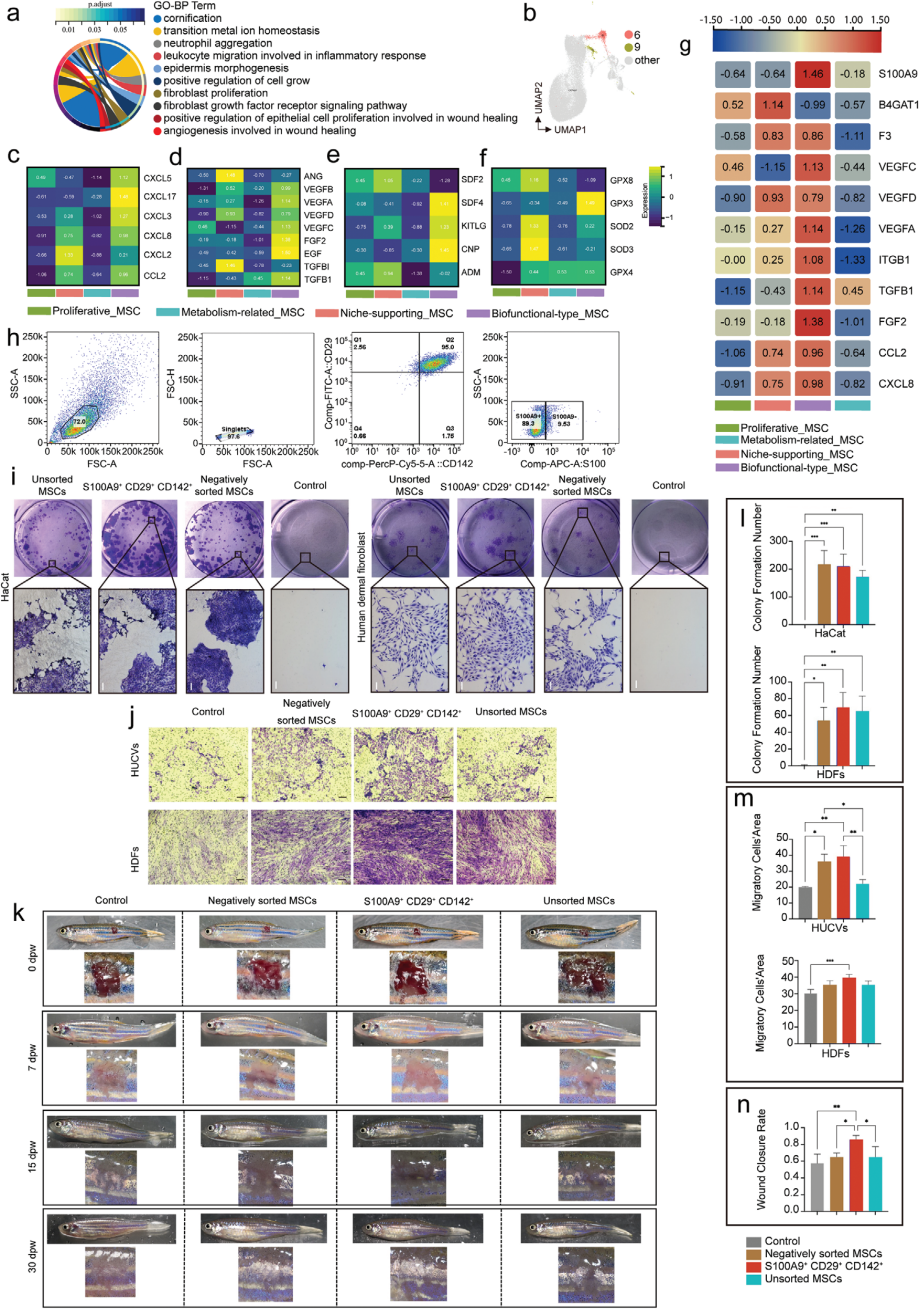
Subpopulation analysis of biofunctional type_MSCs. a) Functional enrichment analysis of biofunctional type_MSCs. b) UMAP visualization of two subsets of biofunctional type_MSCs. c–f) Heatmap showing the relative expression of chemokines, growth factors, stem cell‐specific active factors, and anti‐aging genes in four populations of WJ‐MSCs. g) The expression of wound healing‐related genes in four WJ‐MSC subpopulations. h) FACS‐based sorting strategy to isolate S100A9^+^CD29^+^CD142^+^ subpopulations. i) Colony formation was monitored to evaluate the effect of the S100A9^+^CD29^+^CD142^+^ subpopulations on the proliferation of keratinocytes and fibroblasts. A local enlarged view is shown below. Scale bar: 50 µm. j) Effect of the S100A9^+^CD29^+^CD142^+^ subpopulations on the migration capacity of HDFs and HUCVCs. Scale bar: 50 µm. k) Macroscopic images of zebrafish skin wound healing. l) Quantitative analysis of HaCat and HDFs colony formation number. m) Quantitative analysis of HDFs and HUCVCs migration rate. n) Quantitative analysis of wound healing rate. *p < 0.05, ** *p* < 0.001, *** *p* < 0.0001. HDFs, human dermal fibroblasts, HUCVCs human umbilical vein endothelial cells (HUVECs).

Via combining with single‐cell data analysis and functional enrichment, biofunctional type_MSCs were identified as a dominant functional subpopulation with immunomodulatory and tissue repair functions, including wound healing, leukocyte migration involved in the inflammatory response and positive regulation of cell growth (**Figure** **6**a). We further selected soluble stem cell factors (SCFs) for subpopulation heterogeneity analysis (Figure 6c–f; Table S10, Supporting Information). Although these factors were widely expressed in various subpopulations, there were still some differences. Among the four WJ‐MSC subgroups, biofunctional‐type_MSCs highly expressed various immune‐regulatory and tissue repair‐related genes. The results showed that angiogenesis‐related factors produced by paracrine action such as vascular endothelial growth factor (VEGF), angiopoietin (ANG), fibroblast growth factors (FGF) and epidermal growth factors (EGF), which are relatively highly expressed in biofunctional type_MSCs (Figure 6d). Strikingly, stem cell factor (SCF, alias KITLG), a growth factor that plays a role in the survival, differentiation, self‐renewal and activation of hematopoietic stem cells,^[^ ^21^ ^]^ and stem cell derived factor (SDF) also displayed high expression in biofunctional‐type_MSCs (Figure 6e), suggesting of potential clinical application of MSC subset therapy.^[^ ^22^ ^]^ At the same time, we also showed the expression of anti‐aging genes (Figure 6f), and found that some genes such as SOD2, SOD3 and GPX8 were expressed slightly higher in the niche‐supporting_MSCs than that in biofunctional‐type_MSCs, suggesting the possible interaction between the two subpopulations. The expression similarity of these genes led us to speculate that there was a possible interaction between the two subpopulations.

Our results revealed two distinct clusters of biofunctional‐type_MSCs (cluster 6 and cluster 9; Figure 6b; Figure S11a, Supporting Information). The data demonstrated that increased levels of chemokines, such as CXCL16, CXCL5, CXCL 2 and CXCL3 in cluster 9, revealing immunomodulatory therapeutic properties (Figure S11b, Supporting Information).^[^ ^23^ ^]^ Functional enrichment analyses also revealed distinct functional characteristics between the two subtypes (Figure S11d,e, Supporting Information). In addition, the DEGs of this subpopulation (P3 versus P0) were mainly involved in angiogenesis (Figure S11c, Supporting Information). To subsequently sort this subpopulation for functional verification, we evaluated the expression of marker genes and wound healing‐related genes such as F3 (also known as CD142), VEGF and ITBG1 (also known as CD29) in this subpopulation (Figure 6g). Consistent with the above analysis, the results indicated that biofunctional‐type_MSCs could be the dominant population for wound healing in future clinical applications.

To enrich high‐purity biofunctional‐type_MSCs, we must build a multiple marker gene signature on the basis of the phenotype definition. We first chose the top gene, S100A9, as one of the marker genes (log fold change = 1.578, *p* adjusted value = 1.758E‐68) to ensure the purity of sorting. S100A9 is a cell‐surface marker gene and a protein coding gene involved in the control of inflammatory processes and immunological responses.^[^ ^24^ ^]^ Second, CD29 was identified as another one of the candidate gene, as it is essential for wound healing, re‐epithelialization and vascularization^[^ ^25^ ^]^ and has been shown to be a highly expressed gene of the MSC subpopulation beneficial to wound repair.^[^ ^26^ ^]^ CD142^+^WJ‐MSCs also have potential for wound repair.^[^ ^11a^ ^]^ Finally, based on comprehensive analysis of single‐cell data, gene function data and subcellular localization data, as well as a literature review, we identified CD142, CD29 and S100A9 as the candidate marker genes of this subpopulation for subsequent experiments. Together, these obtained data indicate that the P3 generation of biofunctional type_MSCs may be the dominant functional subpopulation for future clinical applications in wound repair at the single‐cell level.

Based on the above sorting strategy, S100A9^+^CD29^+^CD142^+^ cells accounted for ≈9.53% of cells (Figure 6h). Those cells that did not express these genes or did not express all three genes concurrently were named “negatively sorted MSCs”. All cells grew well and met the requirements of subsequent functional experiments.

We first sought to assess whether S100A9^+^CD29^+^CD142^+^ cells can enhance the proliferative capacity of keratinocytes and fibroblasts. The results showed that after 2 weeks in culture, colonies with a round packed morphology were formed, which displayed a scattered distribution (Figure 6i,l). The number of cells and clones in the control group was markedly lower than that in the other three MSC groups in gross and microscopic examinations (*p* < 0.05). We found that the colonies in the S100A9^+^CD29^+^CD142^+^ group were more compact, although there was no significant difference in cloning capacity among the unsorted MSC, S100A9^+^CD29^+^CD142^+^ MSC and negatively sorted MSC groups. In addition, we found that fibroblasts have a slightly weaker clone‐forming capacity than keratinocytes, which may be determined by their cellular properties. These results showed that the proproliferative capacity of S100A9^+^CD29^+^CD142^+^ cells was not lower than that of unselected MSCs.

Next, to determine whether the S100A9^+^CD29^+^CD142^+^ subpopulation can influence the migration of wound‐healing cells, human dermal fibroblasts (HDFs) and human umbilical vein endothelial cells (HUVECs) were cocultured with three different types of MSCs for 16 h, respectively. The results indicated that the S100A9^+^CD29^+^CD142^+^ subpopulation had a stronger effect on migration than the other groups (Figure 6j,m; *p* < 0.05).

Finally, we evaluated the therapeutic efficacy of the S100A9^+^CD29^+^CD142^+^ subpopulation by using a zebrafish skin wound model. After 1 week of adaptive feeding, we used a sterile scalpel blade to make a 3 mm × 3 mm wound, and then each group of zebrafish was injected subcutaneously with 10 µL of cells (1 × 10^4^ cells) or PBS at the edge of the wound. A small amount of epithelialized tissue was visible on the wound surface after 1 week, and gradually increased over time (Figure 6k,n). On the 30th day after the operation, the wound in the control group was still apparent, with thin epithelial tissue, poor continuity and poor reconstruction of silver stripes. Different degrees of wound healing could be observed in the three MSC treatment groups. Remarkably, epithelization in the S100A9^+^CD29^+^CD142^+^ group was more obvious, and the silver stripes were well reconstructed, and the melanin spots formed by melanocytes around the wounds extended to the wound area. As expected, the highest healing rates of cutaneous wounds were induced by the S100A9^+^CD29^+^CD142^+^ subpopulation in all administration groups (0.857 ± 0.050), and this difference was statistically significant (*p* < 0.05). The healing rates in the control, negatively sorted MSC and unsorted MSC groups were 0.575 ± 0.110, 0.648 ± 0.129 and 0.646 ± 0.129, respectively. These results suggest that S100A9^+^CD29^+^CD142^+^ WJ‐MSCs promote wound healing better than “traditional” WJ‐MSCs. At the same time, these data also reflect the potential value of S100A9^+^CD29^+^CD142^+^ WJ‐MSCs as new drugs.

### 2.9 Spatial Mapping of the Regional Heterogeneity of WJ

**Figure 7:**
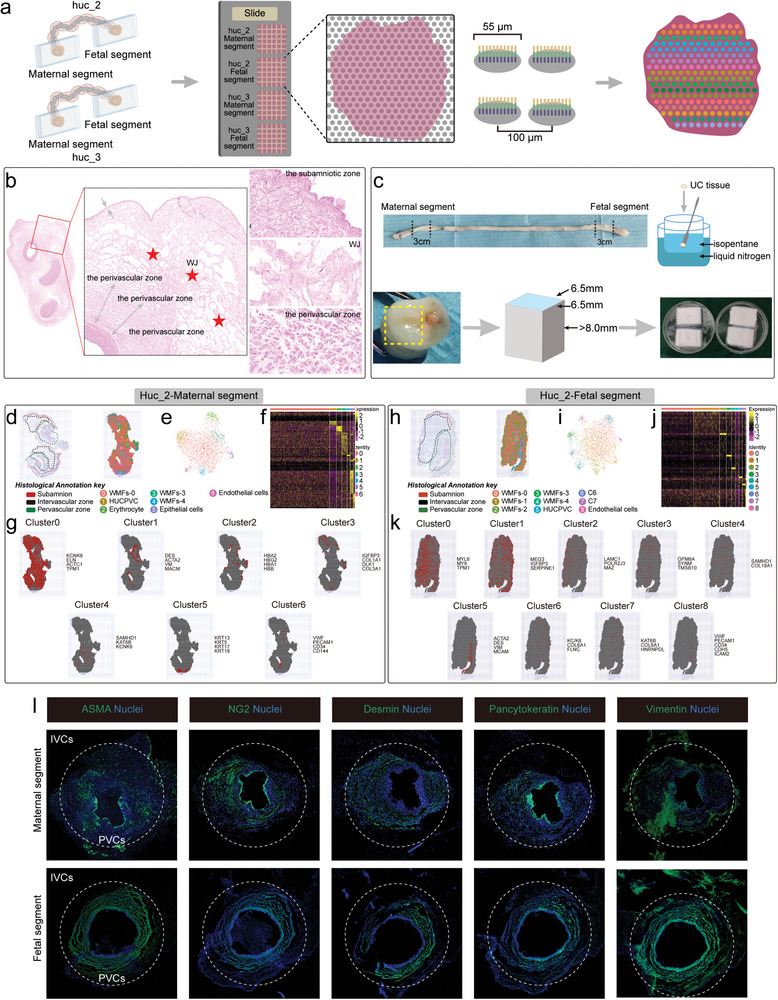
ST analysis of UC tissue. a) Spatial transcriptome sequencing process. b) HE staining of UC tissue. The red pentagonal star indicates the WJ area. c) Preparation of WJ tissue blocks for ST sequencing. d) Spatial distribution and definitions of cell types in the huc_2 maternal segment. e) UMAP projection of cells huc_2 maternal segment. f) Gene expression heatmap of the huc_2 maternal segment. g) Spatial expression pattern of cell types in the huc_2 maternal segment. Red represents gene expression. h) Spatial distribution and definitions of cell types in the huc_2 fetal segment. i) UMAP projection of cells huc_2 fetal segment. j) Gene expression heatmap of the huc_2 fetal segment. k) Spatial expression pattern of cell types in the huc_2 fetal segment. l) Immunofluorescence localization of the HUCPVC subpopulation in the fetal segment and maternal segment. The inner area of the dashed circle indicates the perivascular cell (PVCs) region, and outer dashed circle represents the intervascular cells (IVCs) region.

The human UC can be divided into three regions: the subamniotic zone, WJ, and the perivascular zone (**Figure** **7**b). MSCs with different functions could be present in different parts of the gelatinous WJ;^[^ ^13b^ ^]^ thus, a better understanding of the spatial organization of the cell types present in WJ and the spatial global gene expression heterogeneity of these cells is essential for clinical translation. To this end, we carried out a ST analysis using four slices from different parts of the same UC (huc_2 and huc_3) and matched scRNA‐seq samples (Figure 7a). WJ tissue containing vessels was cut initially from maternal segments and fetal segments of the UC (each 3 cm in length; Figure 7c). After hematoxylin and eosin (HE) staining and the time of permeabilization determination, we annotated the slide for distinct histological features (Figure 7d,h). The numbers of spots on the huc_2‐maternal segment, huc_2‐fetal segment, huc_3‐maternal segment, and huc_3‐fetal segment are shown in Figure S12, Supporting Information.

Next, we associated spatial (spot) gene expression profiles of both segments with physical local information on the tissue images. Several spatial clusters were identified based on cluster‐specific marker genes from four sample sections (Figure 7d–f,h–j; Figure S13a–c,e–g, Supporting Information). WJ‐derived myofibroblasts (WMFs) mapping to the intervascular space were marked by COL4A2 and MYL6, consistent with the gross histologic UC architecture and their anatomical position. Epithelial cells (KRT13, KRT5, and KRT17), endothelial cells (PECAM1, CD34, VWF, and CDH5), and erythrocytes (HBA2, HBG2, HBA2, and HBB) were also identified (Figure 7g,k; Figure S13d,h, Supporting Information). The erythrocyte subpopulation may come from residual blood. Notably, human UC perivascular cells (HUCPVCs) located in the perivascular region have been considered as a source of mesenchymal progenitors^[^ ^27^ ^]^ and mediate communication among various perivascular cell types.^[^ ^28^ ^]^ DES, CD146, VIM, NG2 and ACTA2 were selectively expressed in this cluster,^[^ ^29^ ^]^ and we confirmed these results by immunofluorescence staining (Figure 7l). Pan‐cytokeratin was predominantly expressed in the perivascular region rather than the intervascular stroma, which suggested that HUCPVCs are more differentiated than WMFs.^[^ ^29c^ ^]^ DES, NG2 and ASMA are also predominantly expressed in the perivascular region, with less intervascular expression. VIM is widely expressed in perivascular and intervascular regions, which indicates the similarities and differences in cytoskeletal protein expression in different regions of WJ tissue, and also confirms the accuracy of our analysis of cell types.

**Figure 8:**
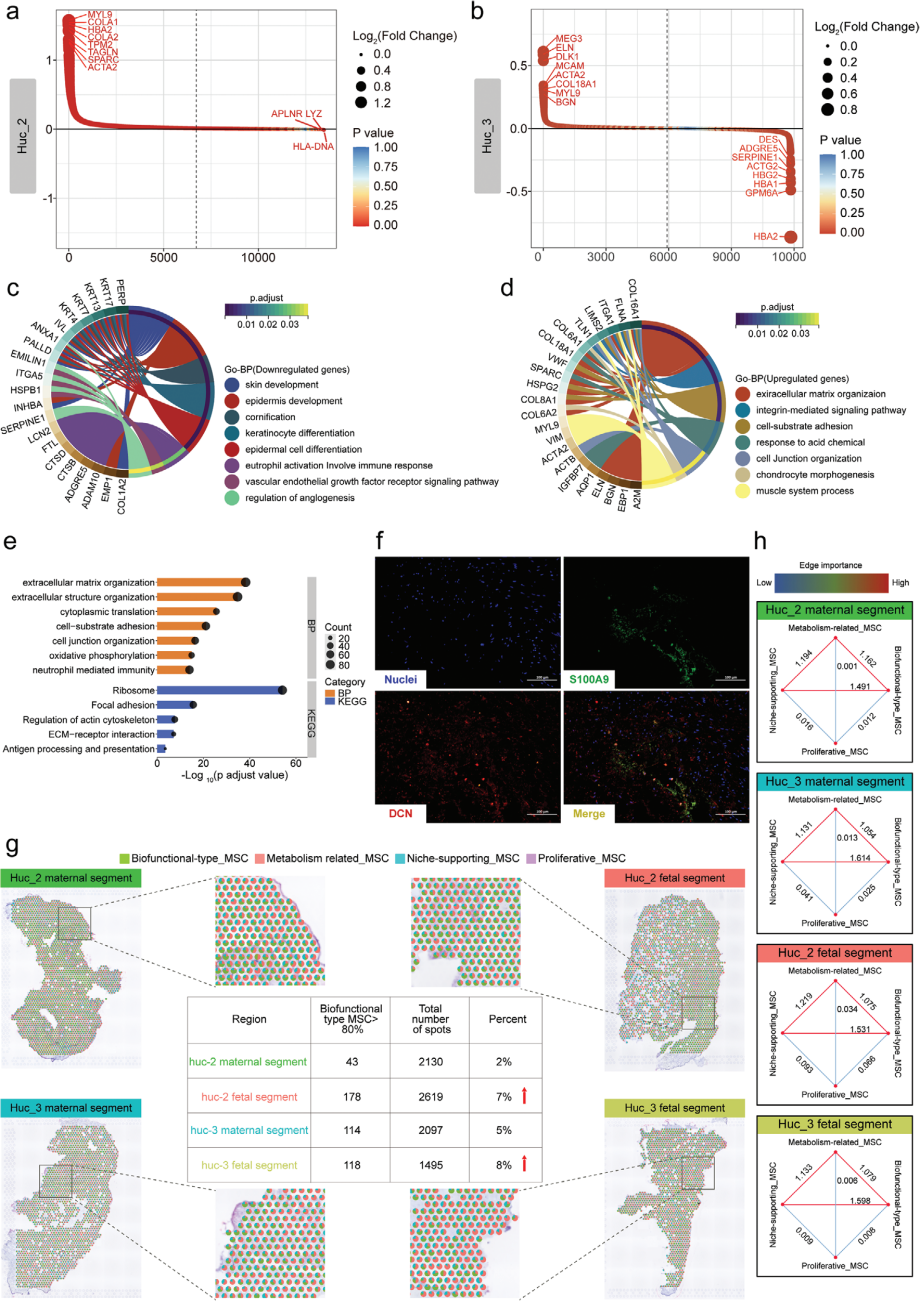
Integrated analysis of scRNA‐seq with ST data. a,b) DEGs between the maternal and fetal segments. Genes were ranked according to LogFC. LogFC > 0.1 and *p* < 0.05 were used to identify significantly upregulated genes. c) GO analysis of downregulated DEGs in the huc_3 fetal segment. d) GO analysis of upregulated DEGs in the huc_3 maternal segment. e) Functional enrichment analysis of huc_2 DEGs. f) The immunofluorescence staining showed co‐localization of the niche‐supporting_MSCs and biofunctional‐type_MSCs. g) Visualization of the proportion of cell types identified by scRNA‐seq in different areas of the UC. The colors represent cell types, and the pie chart shows the percentage of cell types present in each spot. The number of Biofunctional‐type_MSC accounted for more than 80% of one spot in each sample as shown in the table. h) Spatial interaction information for different cell types. Each red dot represents a cell type. The redder the connection color between cell types is, the higher the probability of these two cell types appearing in the same spot. The bluer the color is, the smaller the probability of these two cell types appearing in the same spot. The number represents the value of edge importance.

It is also worth mentioning that there are some differences in cell types and morphology among the four tissue sections (Figure 7d,h; Figure S13a,e, Supporting Information), and we speculated that the underlying reasons are the technical limitations of ST and the characteristics of WJ tissue (i.e., a typically loose fibrous structure and the presence of clefts).^[^ ^30^ ^]^ We next uncovered molecular heterogeneity in the two regions. The results of DEG ranking revealed that there were different transcriptional characteristics between the maternal segment and the fetal segment (**Figure** **8**a,b). Several ECM‐associated genes, such as COL1A1, COL1A2, COL18A1, BGN and ELN, were expressed more highly in the maternal segment than in the fetal segment. Functional enrichment analysis of these DEGs revealed clear enrichment in the ECM organization, cell junction organization, focal adhesion, and ECM‐receptor interaction (Figure 8d,e). In contrast, the fetal segment was enriched in gene sets associated with skin development, epidermis development and neutrophil activation involved in the immune response and regulation of angiogenesis (Figure 8c), which suggested that this segment is a critical MSC source for healing skin wounds. Additionally, the expression of DLK1, which has been reported as a major inhibitor of adipogenesis, was consistently higher in the maternal segment than in the fetal segment (Figure S14a,b, Supporting Information).^[^ ^31^ ^]^ This illustrates that WJ‐MSCs derived from the maternal segment may provide a better model of therapeutic intervention for obesity and related diseases. Collectively, these data revealed the presence of molecular and functional heterogeneity in different UC regions, and provided a representation of cellular components in UC tissue.

### 2.10 Multimodal Identification of WJ‐MSCs by Single‐Cell and Spatial Transcriptomics Analysis

Next, we used SPOTlight to integrate the single‐cell and spatial datasets.^[^ ^32^ ^]^ The percentages of each cell type within each spot for WJ tissue were determined and visualized using pie charts. The results show that there was often more than one cell type in each spot (Figure 8g), which also illustrates the limitations of ST techniques and the importance of multiple omics integration. We counted the number of spots where biofunctional type_MSCs accounted for more than 80% in each spot in each sample. The results showed that the proportion of this subpopulation in the fetal segment was higher than that in the maternal segment (Figure 8g). In addition, spatial interaction analysis between cell types was conducted to explore the frequency of two different cell types that being present in the same spot (Figure 8h). Our results showed that the cell types in different UC regions from the same donor interacted differently, and there were also different interaction relationships for the same UC region among different donors, indicating spatial cellular heterogeneity. Intriguingly, there was close spatial interaction between niche‐supporting_MSCs and the biofunctional‐type_MSCs, and the trend was consistent in all samples (Figure 8h). In addition, the co‐localization of two subpopulations in WJ tissue was also confirmed by immunofluorescent staining (Figure 8f). Overall, the relationship between the function and spatial distribution of cell subpopulations revealed by combined scRNA‐seq and ST analysis is of particular value for evaluating the treatment response of diseases. This also has potentially broad implications for clinical translation and MSC drugs development.

## 3 Discussion

To date, clear evidence for reproducible and standardized MSC therapies is still lacking,^[^ ^33^ ^]^ due to the heterogeneous in the potency of the MSC products.^[^ ^4^, ^5^, ^34^ ^]^ Adequate rational and multilevel systematic research on the identification markers network, gene expression patterns, and biological functions of WJ‐MSCs is urgently needed. Here, we define the cellular heterogeneity of these cells at three levels. First, bulk RNA‐seq was employed to reveal cellular transcriptome heterogeneity at the average expression level. Second, we applied scRNA‐seq to map a single‐cell transcriptomic atlas of WJ‐MSCs from different donors at different passages and, in parallel, combined these data with ST data to reveal the spatial locations of cell subpopulations. The integration of multiple data modalities compensates for the technical limitations of the individual approaches. Our data reveal the cellular and spatial heterogeneity of WJ‐MSCs and identify biofunctional type_MSCs (marked by S100A9^+^CD29^+^CD142^+^) with enhanced wound healing properties. Importantly, by applying SPOTlight, we clarified that this subpopulation is more enriched in the fetal segment of UC tissue. Taken together, these observations support heterogeneity mapping of WJ‐MSCs based on single‐cell information and the spatial context, allowing us to establish protocols for cellular molecular subtyping, and may also facilitate the development of precision WJ‐MSC subpopulation transplantation strategies for wound healing.

Although the recent studies has characterized the MSC (such as BM‐MSCs, AD‐MSC, and UC‐MSCs) heterogeneity and complexity by scRNA‐seq,^[^ ^10^, ^11^, ^35^ ^]^ but different sources of MSCs and different focuses for research can lead to some degree of bias in cellular identity. For example, based on the expression of differentiation genes, Hou et al. reported three UC‐MSC subpopulations, namely osteo‐MSCs, chondro‐MSCs and adipo/myo‐MSCs, which showed pronounced differences in differentiation potentials.^[^ ^35c^ ^]^ Based on the expression patterns of previously characterized bone marrow stroma genes, Wolock et al. defined specific subpopulations in non‐hematopoietic bone marrow cells, including multipotent stromal cells, adipocyte progenitors and pre‐adipocytes, osteoblast‐chondrocyte progenitors, pre‐osteoblast‐chondrocytes, pro‐osteoblasts and pro‐chondrocytes.^[^ ^35a^ ^]^ Our taxonomy is also based on gene expression with annotations of putative functions, while this study does not focus on the differences in the differentiation ability of subpopulations, but instead focuses on the biological functions of subpopulations, so as to pave the way for the subsequent sorting of target functional subpopulations for precision treatment of diseases. Thus, the MSC subpopulations identified in this study are different from previous reports.

Presently, the inherent heterogeneity of WJ‐MSCs at multiple levels has yet to be comprehensively elucidated. By leveraging the advantages of scRNA‐seq, we dissected the distinct molecular profiles within cultured WJ‐MSC populations from different donors. Via combined single‐cell analysis of function and gene expression, four subpopulations were identified, namely proliferative_MSCs (high proliferative potential), niche‐supporting_MSCs (enriched in ECM‐associated molecules), metabolism‐related_MSCs (associated with metabolic ability) and biofunctional type_MSCs (proregenerative and immunomodulatory roles); some of these key molecules were previously implicated in the biologic properties of WJ‐MSCs (e.g., MKI67, FOS, IL‐6, DCN, and SDF4; Figure 2f). Moreover, the results of bulk RNA‐seq broadened our understanding of gene expression patterns and confirmed the technical advantages of scRNA‐seq. Among the four candidate WJ‐MSC subpopulations, three are particularly interesting: 1) proliferative_MSCs (62.00%) are the most abundant population and are critical for cellular growth and development, which is in line with the consensus that MSCs possess high proliferative capacity in an in vitro culture environment.^[^ ^36^ ^]^ Indeed, pathway enrichment analysis revealed that this subpopulation is involved in several of the above processes. Importantly, we mapped cells transition paths from proliferative_MSCs to other MSC subpopulation, indicating that highly proliferative cells begin a program of cell‐state transition after completing its cell cycle;^[^ ^37^ ^]^ 2) cell‐matrix dynamic interactions and ECM remodeling have been considered to significantly affect the integrity of the stem cell niche.^[^ ^38^ ^]^ We found that niche‐supporting_MSCs acted as the central hub of communication for the MSC populations, suggesting that they play a role in orchestrating different aspects of MSC function. Expectedly, there was a close spatial interaction between the niche‐supporting_MSC and the biofunctional‐type_MSC from the result of ST. In addition, these cells also exhibited high osteogenic and chondrogenic differentiation potential, which was confirmed by the high expression of COL1A1 and COL11A1 in niche‐supporting_MSCs compared to other subpopulations;^[^ ^39^ ^]^ The observations are consistent with previous reports that different UC‐MSC subpopulations displayed distinct differentiation capacity;^[^ ^11^, ^35^ ^]^ and 3) notably, biofunctional type_MSCs highly expressed immune‐related and proangiogenic genes. The enrichment of GO terms such as “angiogenesis involved in wound healing” and “leukocyte migration involved in inflammatory response” suggested the potential of these cells for therapeutic strategies.

What is the molecular basis for the different functions of subpopulations, and what are the implications of these differences? Previous studies have shown that the functional phenotype of MSC subpopulations is exquisitely controlled by the upstream TF regulatory network.^[^ ^40^ ^]^ Through TF prediction analysis, we identified several TFs associated with MSC growth, development, metabolism and differentiation, such as E2F2 and E2F7 (proliferative_MSC), STAT2 (niche‐supporting_MSC), SP4 (metabolism‐related_MSC) and KLF3 (biofunctional type_MSC).^[^ ^41^ ^]^ Strikingly, we also predicted two previously unreported TFs, ELK3 and RREB1, as important drivers of biofunctional type_MSCs to achieve wound repair.^[^ ^42^ ^]^ Taken together, these findings allowed us to construct a molecular framework for identifying WJ‐MSCs from different sources as a complement to previous knowledge, and they provide a new paradigm for the involvement of MSC subpopulations in tissue repair/remodeling.

Obtaining effective functional MSC subpopulations is of great significance for constructing tissues or organs in vitro, developing new drug vectors and treating various diseases in the clinic. To support our hypothesis, we purified biofunctional type_MSCs according to cell‐surface marker genes and representative pro‐angiogenic genes (S100A9^+^CD29^+^CD142^+^ cells correspond to biofunctional type_MSCs) to confirm their potency in wound healing. In the in vitro results showed that S100A9^+^CD29^+^CD142^+^ MSCs had a beneficial effect on wound‐healing cells (keratinocytes/fibroblasts/endothelial cells), promoting cellular proliferation and migration. Similar findings were observed in a zebrafish skin injury model. The quality of wound healing in zebrafish treated with this subpopulation was observed to be better than that of zebrafish treated with unsorted MSCs, as indicated by the healing rate and the accelerated re‐epithelialization process.

MSCs are the ideal seed cells for cell therapy to repair skin wounds.^[^ ^43^ ^]^ Although the molecular mechanisms of these phenomena are still unclear, the paracrine activity of MSCs is believed to result in the stimulation of angiogenesis and the recruitment of local stem cells;^[^ ^44^ ^]^ this paracrine activity includes the secretion of VEGFA,^[^ ^45^ ^]^ F3,^[^ ^46^ ^]^ TGFB1,^[^ ^47^ ^]^ CCL2,^[^ ^48^ ^]^ FGF2,^[^ ^49^ ^]^ ITGB1,^[^ ^50^ ^]^ PDGF, IL‐6, and MCP‐1.^[^ ^51^ ^]^ Our single‐cell data suggest that many angiogenesis‐related genes are highly enriched in biofunctional type_MSCs. Furthermore, it is well established that ligand–receptor complexes play critical roles in tissue regeneration and wound closure.^[^ ^52^ ^]^ For instance, the interaction of the AXL receptor and its ligand GAS6 was observed in biofunctional type_MSCs and niche‐supporting_MSCs, which is consistent with previous research showing that AXL‐GAS6 contributes to restoring the integrity of the endothelial barrier and promoting epithelial cell survival in the context of vessel damage.^[^ ^53^ ^]^ The FDZ6‐WNT5A interaction, which functions in angiogenesis, was enriched in biofunctional type_MSCs and proliferative_MSCs.^[^ ^54^ ^]^ Thus, in conclusion, our data strongly suggest that S100A9^+^CD29^+^CD142^+^ cells exhibit excellent wound reparative function both in vivo and in vitro.

WJ‐MSCs are considered an excellent candidate source for tissue engineering.^[^ ^55^ ^]^ However, little is known about which segment of the UC is the best optimal treatment option for specific diseases.^[^ ^13^, ^56^ ^]^ To address this question, we first elaborated on previously unstudied spatial‐gene expression profiles in different regions of WJ tissue by using ST. Consistent with previous reports, we identified cell types of the UC in situ, including WMFs, HUCPVCs, epithelial cells and endothelial cells.^[^ ^29^, ^57^ ^]^ The distribution of cell types within a single segment was consistent with anatomical annotations. For example, WMFs are located in the intervascular zone, while HUCPVCs can be observed in the perivascular zone. Immunofluorescence also confirmed the presence and spatial distribution of HUCPVCs. In addition, varying expression abundance was found between different segments, implying substantial spatial heterogeneity.^[^ ^56^ ^]^ We were surprised to find that biological processes related to angiogenesis and wound repair were significantly enriched in the fetal segment, while the maternal segment was more enriched in ECM organization.

To increase the resolution of our ST data, we then integrated it with scRNA‐seq data by utilizing SPOTlight. From the spatial distribution characteristics of WJ‐MSC subpopulations obtained from scRNA‐seq data, we found that there was a low proportion of proliferative_MSCs, but the majority of WJ‐MSCs are proliferative_MSCs based on the scRNA‐seq data. The reasons for this are severalfold. First, MSCs in long‐term culture show a strong proliferative ability,^[^ ^58^ ^]^ and second, explant method used in our study can harvest pure and less heterogeneous cells with higher cell viability and number when compared with enzymatic method.^[^ ^59^ ^]^ Third, changes in microenvironment of stem cells in vivo and in vitro will cause changes in gene expression. As a starting point, proliferative_MSCs can transition into the other three types of WJ‐MSC subsets, so they may exist as “seed cells” with a small proportion in vivo. Once out of the niche, the original gene expression activity associated with stem cell niche support may be lost,^[^ ^60^ ^]^ the cellular state transformation between WJ‐MSC subpopulations will increase the proportion of proliferative_MSCs in vitro.

In addition, we found that biofunctional‐type_MSCs tend to be dominant in the fetal segment compared with the maternal segment, suggesting that the biofunctional type_MSCs isolated from the fetal segment might be a preferred source for wound repair. We also found similar expression of anti‐aging related genes in the niche‐supporting_MSCs and biofunctional‐type_MSCs, and they had strong co‐localization spatially, indicating that combined treatment of the two subpopulations in the field of anti‐aging may have unexpected results. The use of specific subpopulations of MSCs in tissue regeneration is an emerging idea and represents an innovative approach; nevertheless, further analysis is necessary to corroborate and extend this conclusion.

Overall, our study mapped the first comprehensive large‐scale spatial and single‐cell transcriptomic atlas to decipher the heterogeneity of WJ‐MSCs and elucidate their architecture from multiple angles. Linking the molecular characteristics of MSCs to their function is extremely challenging. Our data will be an important resource for future similar studies. Of note, we also revealed a critical WJ‐MSC subpopulation with wound repair capacity and suggested that this subpopulation derived from the fetal segment of the UC may have a desirable therapeutic potency. Thus, this research has enhanced the value of the WJ‐MSC subpopulation as a resource, promoting the development of new treatment strategies. Importantly, this information has significant implications for clinical translation and drug development.

This study has several limitations. First, although we established a strategy for molecular typing of WJ‐MSCs based on characteristic genes, the candidate genes identified need to be further validated with functional experiments. Second, the morphology of the WJ specimens prepared for ST was inconsistent, and the gene expression levels were generally low, which limited our ability to evaluate spatial features. There are two causes of these limitations: 1) the jelly‐like characteristics of the WJ make it difficult to carefully cut the UC into 6.5 × 8.5 × 8.0 mm segments in the shortest possible time; and 2) there are many cracks between the fiber structures of the UC, resulting in RNA degradation. Future work will use other techniques that are more suitable for this tissue, such as 10× Visium FFPE. Third, many aspects behind S100A9^+^CD29^+^CD142^+^ WJ‐MSCs treatment response mechanisms remain to be elucidated. In the future, the combination of bulk RNA‐seq and scRNA‐seq will be used to investigate the molecular signaling pathways which are involved in the repair of zebrafish skin wound. Finally, we cannot ignore the possibility of poor cell activity, low yield, cell phenotype changes, and subpopulation impurity brought about by FACS sorting.

## 4 Experimental Section

### Human UC Sample Collection

UCs were obtained from Fujian Medical University Union Hospital (*n* = 3). Ethical approval was granted by the Ethics Committee of Fujian Medical University Union Hospital (2020KY0117). All healthy donors signed the written informed consent form.

### WJ‐MSC Culture

Human WJ‐MSCs were cultivated by explant cultures. After the UC was collected and tested according to quality control standards, the cord was sterilized with 75% alcohol, placed in a biological safety cabinet, washed with saline, and cut into 2 cm pieces. Then, the veins and arteries were removed, and WJ was harvested and cut into pieces 1 mm^3^ in size. The pieces were added to 150 cm^2^ culture flasks (ThermoFisher), and 25 mL of prewarmed stem cell medium (MSCYF01‐500, MSCYF02‐20, Yinfeng biological group, LTD., China) was added to each flask. The samples were carefully cultured in humidified air with 5% CO_2_ for the first 7 days. After the first 7 days of incubation, the medium was exchanged every 3 days until 80–90% confluency were reached. Cells were dissociated with TrypLE Express (Gibco Life Technologies, 12604039). Primary cells and cells at passage 3 were used for subsequent sequencing analysis and validation experiments.

### Immunofluorescence Staining

Immunofluorescence studies were performed on frozen UC sections. Frozen sections were baked for 10 min at 37 °C before being washed three times for 5 min each in PBS. BSA (3%) was added to cover the tissue to block nonspecific binding. Subsequently, the primary antibody (anti‐alpha smooth muscle Actin, Abcam, ab124964, 1:300; anti‐pan‐Cytokeratin, SANTA CRUZ, sc‐8018, 200 µg mL^−1^; anti‐Desmin, Abcam, ab227651, 1:100; anti‐Vimentin, Abcam, ab92547, 2 µg mL^−1^) was added overnight at 4 °C and washed with PBS. Next, the tissue was covered with secondary antibody and incubated at room temperature for 50 min in the dark. After washing in PBS (3 × 10 min), nuclear staining with DAPI solution (Life Technologies) was performed for 10 min. Spontaneous fluorescence quenching reagent (#G1221, Servicebio) was then added, and the samples were incubated for 5 min and rinsed with tap water for 10 min. Images were acquired under Nikon Eclipse C1 and analyzed with ImageJ software. For WJ‐MSC immunofluorescence, after the WJ‐MSC slides were rinsed three times with PBS and fixed in 4% paraformaldehyde for 15 min, the WJ‐MSCs were blocked with PBS containing 0.3% Triton X‐100 and 5% normal sheep serum for 1 h. Primary antibodies, purchased from Abcam (Anti‐B4GALT1, ab121326; Anti‐Decorin, ab175404; Anti‐HMGB2, ab124670; Anti‐Profilin 1, ab124904), were added overnight at 4 °C. Then, slides were washed 3 times, the second antibody (Goat Anti‐Mouse IgG H&L, ab150115) was diluted in blocking solution and incubated for 1 h. Finally, nuclei were stained with DAPI, and staining was observed by fluorescence microscopy.

### Fluorescence Activated Cell Sorting

For isolation of the S100A9^+^CD29^+^CD142^+^ subpopulation, WJ‐MSCs were stained with fluorescent antibodies targeting the positive selection markers S100A9 (Biolegend, 1:25), CD142 (BD Biosciences, 5 µL/test), and CD29 (BD Biosciences, 5 µL/test) for 30 min at 4 °C. After washing with resuspension buffer at 300 × g for 5 min, the WJ‐MSCs were sorted or analyzed by FACS utilizing a BD FACSAria II.

### Colony Formation Assay

Colony formation was determined by a clonogenic assay using keratinocytes and dermal fibroblasts (all cells were purchased from Procell Life Science & Technology Co. Ltd.). Cells were harvested in the logarithmic growth phase and centrifuged. Then, cells (700 cells/well) were added to the lower chamber of a 12‐well Transwell plate (Corning) in serum‐free medium; each group of MSCs was seeded into the upper chamber. All cells were cultured continuously in a 37 °C incubator with 5% CO_2_ for two weeks. Next, the cells were washed thrice by PBS, fixed with paraformaldehyde, and further stained with crystal violet for 20 min. The dye was slowly washed with running water and dried in air. The number of cell colonies was counted with an SLR camera and microscope (100×).

### Cell Migration Assay

Transwell chamber assays were performed to assess the migration of HDFs or HUVECs. Negatively sorted MSCs, S100A9^+^CD29^+^CD142^+^ MSCs, or unsorted MSCs were pipetted into the upper chamber of a 24‐well plate (2 × 10^5^ cells per well). No cells were inoculated in the lower compartment for the control group. WJ‐MSCs were seeded in the upper chambers (5000 cells per well). After 16 h of treatment, the chamber was fixed and stained with crystal violet. Nonmigrating cells in upper chamber were removed. Stained cells in three random fields were visualized and counted.

### Zebrafish Skin Wound Model

All protocols and procedures involving zebrafish were approved by the Animal Use Committee of Fujian Medical University. Wild‐type male zebrafish of the AB strain were purchased from the Bio‐Service Co. Ltd. (Fuzhou, China). Zebrafish were raised at 28 °C in an automatic fish housing system and were kept on a 14 h light/10 h dark cycle. The zebrafish were fed with freshly hatched Artemia once per day. Adult zebrafish were divided into four groups, the control group (*n* = 6), mixed MSC group (unsorted WJ‐MSCs, *n* = 6), S100A9^+^CD29^+^CD142^+^ MSC group (*n* = 6), and negatively sorted MSC group (*n* = 6). Then, 0.03% tricaine (Shanghai Aladdin Biochemical Technology Co. Ltd. Shanghai, China) was added to anesthetize the zebrafish. Following anesthesia, a full‐thickness wound (diameter ≈3 mm) was created with micro‐ophthalmic scissors onto the left side of the back of the zebrafish. Then, 10 µL of experimental solution was subcutaneously injected into the periwound area using a 33 gauge syringe (Hamilton). The needle was carefully kept in situ for 5 min after injection, and the fish was then returned to the water in the circulating system. If any injection fluid was observed to leak at the injection site, the fish was euthanized and not included in subsequent experiments. At 0, 5, 15 and 30 days post‐wounding (dpw), the fish were anesthetized and subsequently photographed. Wound area was measured using ImageJ software.

### Single‐Cell RNA Sequencing

The cultured WJ‐MSCs (huc_1 P0, huc_1 P3, huc_2 P0, huc_2 P3, huc_3 P0, and huc_3 P3) were digested to single‐cell suspension and adjusted to ≈1.0 × 10^6^ cells/mL. In accordance with the 10× Genomics recommended protocol, scRNA‐seq was performed with the Chromium 3′ V3 Chemistry (10× Genomics). An Illumina Novaseq6000 sequencer was used to sequence the single‐cell RNA‐seq libraries (performed by CapitalBio Technology, Beijing). Raw sequencing data was processed using the cellranger‐3.1.0. Feature‐barcode matrix generation and clustering were performed with Cell Ranger count module through alignment, filtering, and barcode counting.

### Quality Control

10× Genomics‐derived data (huc_1 P0, huc_1 P3, huc_2 P0, huc_2 P3, huc_3 P0, and huc_3 P3) were collected. The downstream analyses using the Seurat R program filtered out cells with gene numbers less than 200 or in the top 1%, and mitochondrial gene ratios greater than 25% (version 3.0).^[^ ^61^ ^]^

### Cell Clustering and Dimensionality Reduction

The Normalization function was used to normalize scRNA‐seq data (normalization.method = “LogNormalize” and scale.factor = 10 000). And then highly variable genes were identified using the FindVariableFeatures function (setting: selection.method = “vst”, nfeatures = 2000). Principal component analysis was then running using significantly variable genes, and Uniform Manifold Approximation and Projection (UMAP) was performed to visualize data using the first 50 principal components (“resolution” value 0.6).

### Dataset Integration

For integration of different data sets and avoiding batch effects, the CCA integration tool was used.^[^ ^62^ ^]^ The FindIntegrationAnchors function was used to identify integration anchors, and then IntegrateData was used to merge the samples into one object (dim = 1:50). Integrated objects were scaled and runPCA.

### DEGs and Enrichment Analyses

The FindAllMarkers or FindMarkers function were used to identify DEGs. Functional annotation enrichment of top 20 marker genes of the cluster were performed using KOBAS software, and R package was used for visualizing the results. To adjust for multiple testing, a Benjamini–Hochberg test was used.

### Pseudotime Analysis

The pseudotime trajectory was determined with the Monocle2 package with the default settings. Three major criteria were used to select target genes: expressed in more than 10 cells, average expression > 0.1, and *q* value < 0.01. The DDRTree method was used for dimension reduction with max_components set at 2, and the cells were ordered using the orderCells function. The differential GeneTest function was used to find DEGs among different clusters. Dynamically expressed genes along pseudotime were clustered with the “plot_pseudotime_heatmap” function. The plot_genes_branched_heatmap function was used to visualize the expression of genes in branched modules.

### SingleR Annotation

Cell type was annotated with singleR. Blueprint_Encode or HPCA was used. Utilizing reference transcriptomic datasets of pure cell types, this method allows unbiased cell type recognition using scRNA data by inferring the origin of each individual cell based on its own transcriptome.

### Cell–Cell Interactions

CellPhoneDB (https://www.cellphonedb.org/, v2.0) was employed to infer potential ligand–receptor interactions.^[^ ^63^ ^]^ scRNA‐seq data from cells annotated as proliferative_MSCs, niche‐supporting_MSCs, metabolism‐related_MSCs, and biofunctional‐type_MSCs were input into CellPhongDB. Only receptor and ligand subclusters with expression levels of more than 10% were considered when determining the most relevant interactions between various cell types. Then, the cell clusters were compared in pairs using the default setting of CellPhoneDB and the number of ligand–receptor complexes was calculated between each pair of clusters (*p* < 0.05).

### SCENIC Analysis

As described previously, SCENIC analysis was carried out.^[^ ^17^ ^]^ The software programs SCENIC27, AUCell, and RcisTarget were used. RcisTarget and AUCell were used to trim modules for targets and assess the regulatory network's activity across all cells, respectively.

### TF Network Construction

TFs were predicted using TFBSTools, JASPAR database, and NetworkAnalyst database (https://www.networkanalyst.ca). Cytoscape software was used to visualize the gene and TF network.

### Bulk RNA‐seq Data Analysis

The quality control experiment and quantification of RNA obtained from P0 and P3 WJ‐MSCs were performed (named as Bhuc_1P3, Bhuc_2P0, Bhuc_3P0, Bhuc_4P0, Bhuc_4P3). Sequencing libraries were carried out by the NEBNext Ultra RNA Library Prep Kit for Illumina (#E7530S,NEB). A Bowtie1 software program was used to map the sequencing reads onto the reference gene set.^[^ ^64^ ^]^ The gene expression profile was generated using the Perl script program, and then differentially expressed mRNAs were identified by edgeR program.^[^ ^65^ ^]^ Significantly differentially expressed genes were identified between the two samples as genes with a *p* value < = 0.05 and |log2FC|> = 1.

### 10× Visium ST—Staining and Imaging

Cryosections (10‐µm) were cut and mounted on GEX arrays. Sections were loaded onto a Thermocycler Adaptor. Following an incubation of 1 min at 37 °C, fix for 30 min in −20 °C with methyl alcohol, the sections were stained with hematoxylin and eosin (H&E). At a resolution of 10×, these brightfield images were scanned on a Leica DMI8 whole‐slide scanner.

### Permeabilization and Reverse Transcription

The Visium spatial Library construction kit (10× Genomics, PN‐1000184) were used to process the data. Slide cassettes were used to create leak‐proof wells to add reagents to the samples. After adding 70 µL of permeabilization enzyme, the mixture was incubated at 37 °C for 12 min. For cDNA synthesis, 75 µL reverse transcription Master Mix was added and then the wells were washed with 100 µL SSC.

### cDNA Library Preparation for Sequencing

Remove RT Master Mix from the wells at the end of first‐strand synthesis. Then, 75 µL 0.08 m KOH was added and incubated for 5 min at room temperature. Next, KOH was removed from the wells, and the wells were washed with 100 µL EB buffer. A total of 75 µL of Second Strand Mix was added to each well for second‐strand synthesis. On an S1000TM Touch Thermal Cycler, cDNA amplification was carried out according to the manufacturer's instructions (Bio Rad). Visium spatial Library construction kit was used to build Visium spatial libraries (10× Genomics, PN‐1000184). Finally, the libraries were sequenced on a Novaseq 6000 (Illumina) at sequencing depth of at least 100 000 reads per spot (performed by CapitalBio Technology, Beijing).

### Processing of the ST Data

To construct a feature‐barcode matrix and locate clusters, the Spaceranger count module was used to perform alignment, filtering, barcode counting, and UMI counting. PCA was used to reduce dimensionality, and the first 10 principle components were utilized to build clusters using the K‐means and graph‐based algorithms, respectively. The other clustering method is Seurat 3.2. PCA was used to reduce dimensionality, and UMAP was used to visualize the result at a resolution level of 0.6.

### SPOTlight

To determine spatial interactions, SPOTlight was used to deconvolve the spatial transcriptome data by combining scRNA‐seq cell type profiles.

### Statistics

All data are shown as the mean ± SD. Statistical analyses were performed with GraphPad Prism version 8. The statistical details for each experiment are provided in the figure legends. A *p* value < 0.05 was considered statistically significant.

## Conflict of Interest

The authors declare no conflict of interest.

## Author Contributions

P.H.C., S.J.T., and M.L. contributed equally to this article. J.H.C., L.W.C., and X.S.C. conceptualized and supervised this study. P.H.C., S.J.T., and M.L. contributed to the study design and performed most experiments. K.L.H., J.L., Q.X.L., and Y.T. were engaged in sample collection and preparation. P.H.C. and S.J.T. contributed to bioinformatics and statistical analysis. D.Z.W., Y.Q.Q., and C.X.C. contributed to data collection and figure design. P.H.C., S.J.T., D.Z.W., and Z.Q.F. were responsible for animal experiments. H.R.Z., H.Q.G., and H.Y.W. performed immunostaining analyses. P.H.C. and S.J.T. drafted the manuscript. S.R.L., J.H.C., L.W.C., and X.S.C. reviewed and prepared the final manuscript. All authors approved the final manuscript.

## Supporting information

Supporting Information

Supporting Table 1

Supporting Table 2‐10

## Data Availability

The data that support the findings of this study are available from the corresponding author upon reasonable request.
