# Supplementary material for: Single‐Cell and Spatial Transcriptomics Decodes Wharton's Jelly‐Derived Mesenchymal Stem Cells Heterogeneity and a Subpopulation with Wound Repair Signatures
Source: Adv Sci (Weinh). 2022 Dec 11;10(4):2204786. doi: 10.1002/advs.202204786 (PMC9896049; doi:10.1002/advs.202204786)
Supplement: Supplementary file 1 — Supporting Information [file ADVS-10-2204786-s003.pdf]

Supporting Information

Single-cell and Spatial Transcriptomics Decodes Wharton's Jelly-derived Mesenchymal Stem Cells Heterogeneity and a Subpopulation with Wound Repair Signatures

Penghong Chen#, Shijie Tang#, Ming Li#, Dezhi Wang, Caixiang Chen, Yiqun Qiu, Zhuoqun Fang, Haoruo Zhang, Hangqi Gao, Haiyan Weng, Kailun Hu, Jian Lin, Qingxia Lin, Yi Tan, Shirong Li, Jinghua Chen, Liangwan Chen*, Xiaosong Chen**

#These authors contributed equally to this article.

* Corresponding author

Dr. Penghong Chen, Dr. Shijie Tang, Dr. Ming Li, Dr. Dezhi Wang, Dr. Caixiang Chen, Dr. Yiqun Qiu, Dr. Zhuoqun Fang, Dr. Haoruo Zhang, Dr. Hangqi Gao, Dr. Haiyan Weng, Dr. Kailun Hu, Dr. Jian Lin, and Prof. Xiaosong Chen

Department of Plastic Surgery and Regenerative Medicine, Fujian Medical University Union Hospital, Fuzhou, 350001, China

Department of Plastic Surgery and Regenerative Medicine Institute, Fujian Medical University, Fuzhou, 350001, China

Engineering Research Center of Tissue and Organ Regeneration, Fujian Province University, 350001, China

Department of Stem Cell Research Institute, Fujian Medical University, Fuzhou, 350004, China

E-mail: chenxiaosong74@163.com

Prof. Liangwan Chen

Department of Cardiac Surgery, Fujian Medical University Union Hospital, Fuzhou, 350001, China

Engineering Research Center of Tissue and Organ Regeneration, Fujian Province University, 350001, China

E-mail: chenliangwan@tom.com

Prof. Jinghua Chen

Department of Pharmaceutical Analysis, the School of Pharmacy, Fujian Medical University, Fuzhou, 350100, China

E-mail: cjh_huaxue@126.com

Dr. Qingxia Lin

Department of Obstetrics, Quanzhou Women and Children's Hospital, Quanzhou, 362000, China

Prof. Yi Tan

Qilu Cell Therapy Technology Co., Ltd, Jinan, Shandong, 250000, China

Prof. Shirong Li

Department of Plastic and Reconstructive Surgery, Shinrong Plastic Surgery Hospital, Chongqing, China

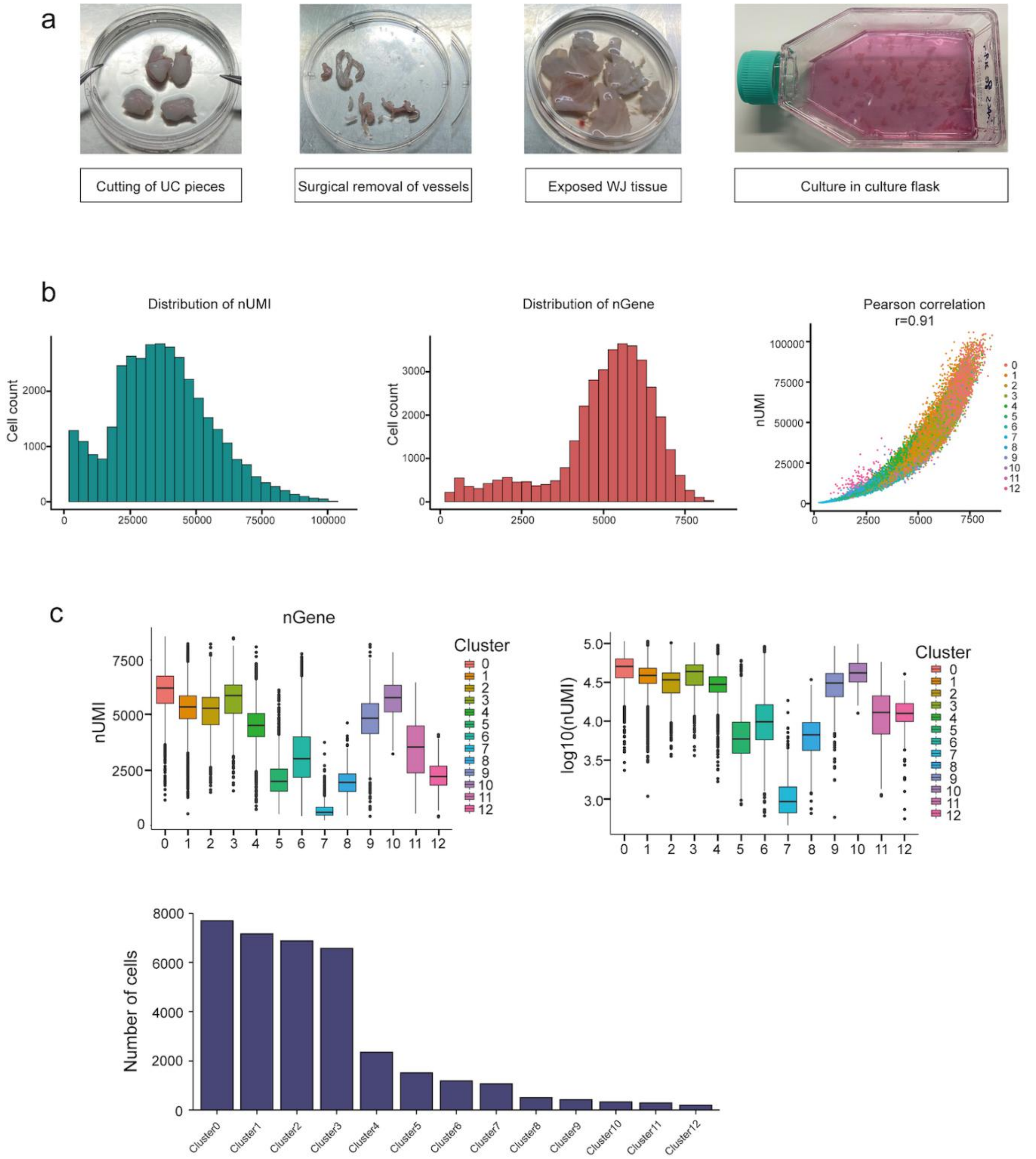

Figure S1. The extraction process of WJ-MSCs and scRNA-seq data quality assessment. **a)** WJ-MSCs were isolated from WJ. **b)** The Pearson correlation between nUMI and nGene, and the histogram showing the distribution of nGene and nUMI. **c)** The boxplots and histogram showing the number of UMI, nGene and cell across different clusters.

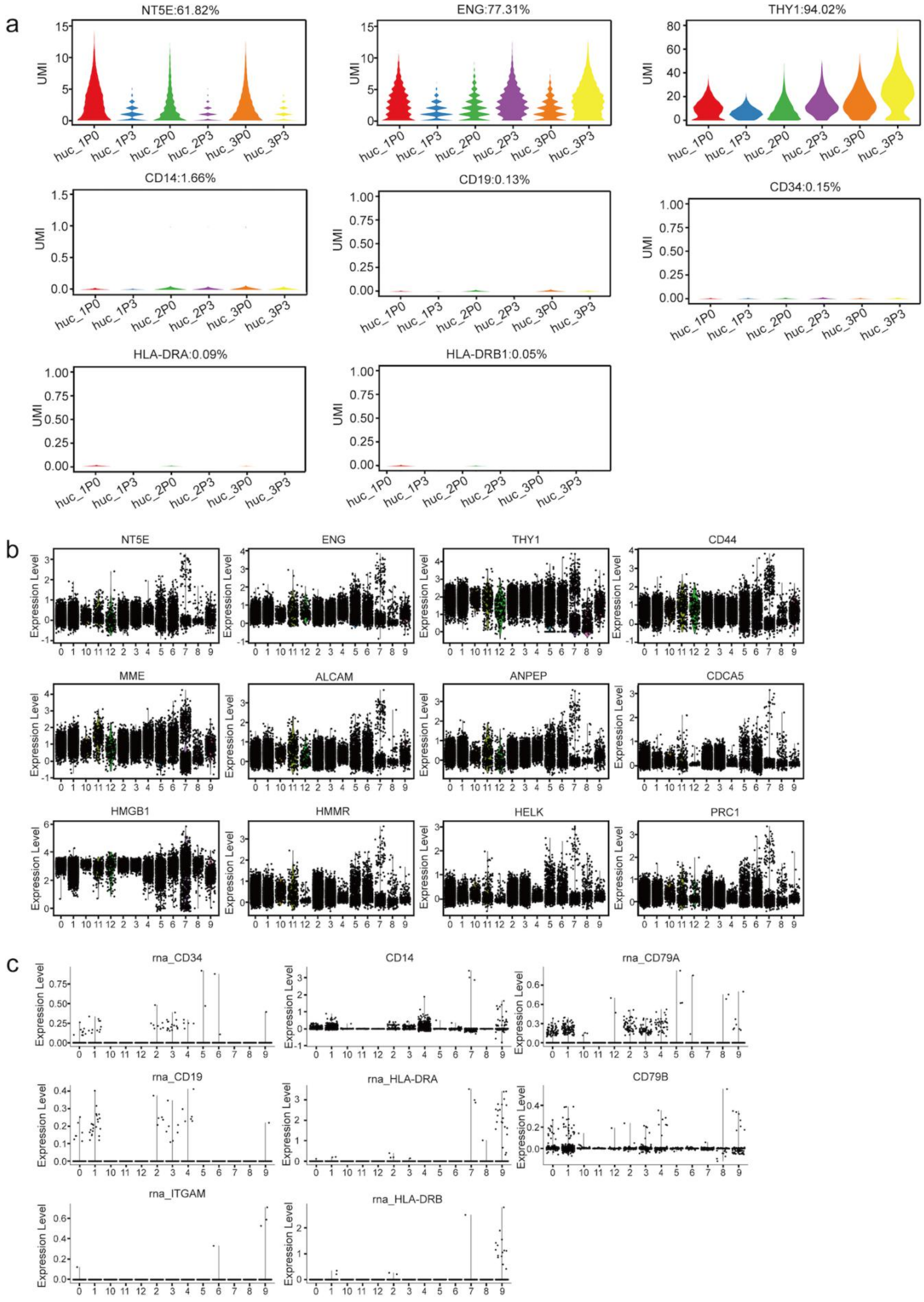

Figure S2. Expression of MSCs classically expressed genes in 6 single cell samples. a) The violin plot showing expression of MSC-positive genes (NT5E, ENG, and THY1) and MSC-negative genes (CD14, CD19, CD34, HLA-DRA, and HLA-DRB) in the different

samples. The number represents the percentage of cells with at least one UMI. **b)** The violin plot showing the expression of classical MSC postive markers in all culsters. **c)** The violin plot showing the expression of classical MSC negative markers in all culsters.

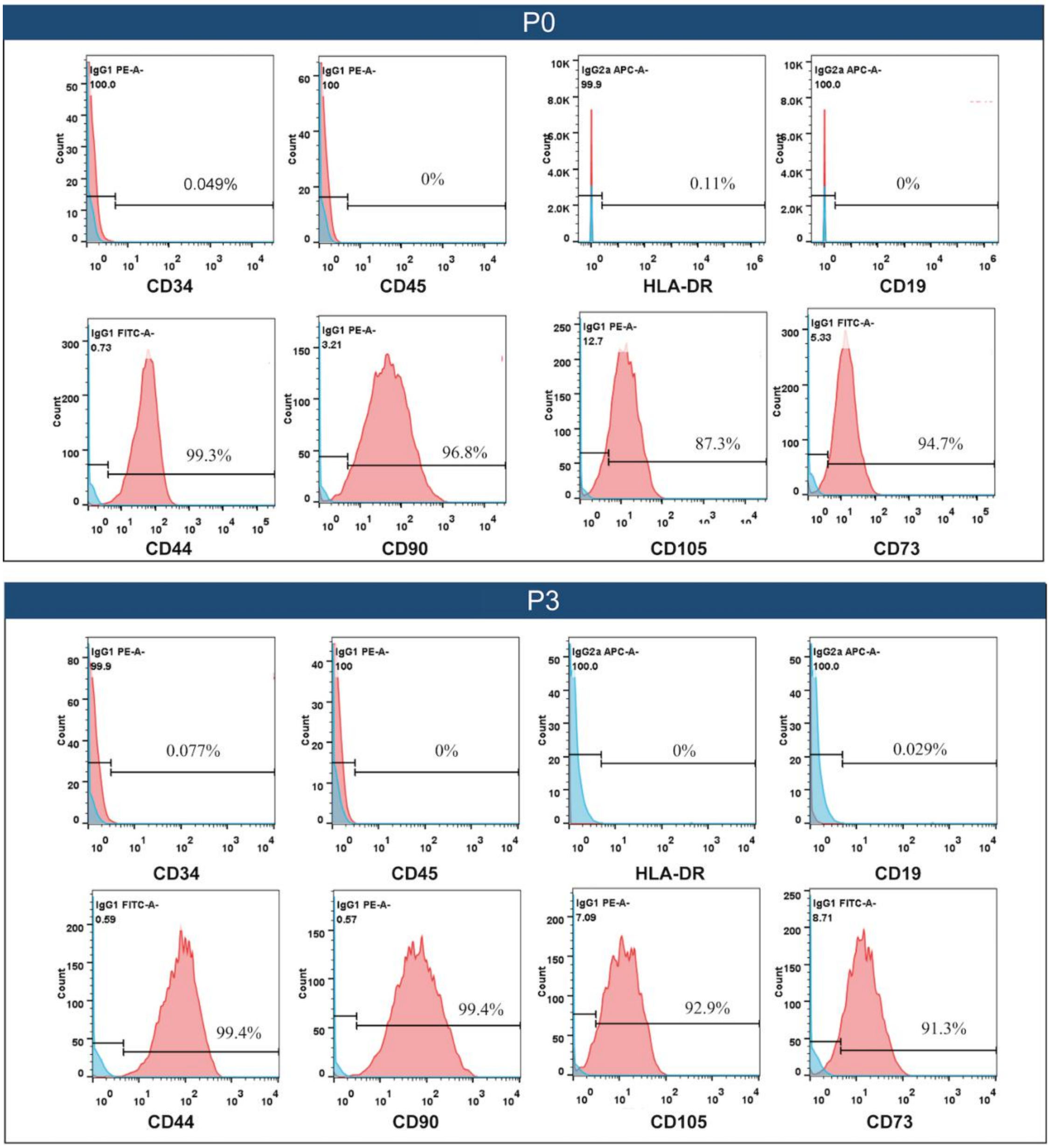

Figure S3. Immunophenotyping of WJ-MSCs in P0 and P3 passage. MSCs surface markers CD44, CD73, CD105, and CD90 were highly expressed in P0 and P3, while hematopoietic cell surface markers CD34, CD45, CD19, and HLA-DR were all lowly expressed. Blue indicates the homotype control, and red indicates the surface marker.

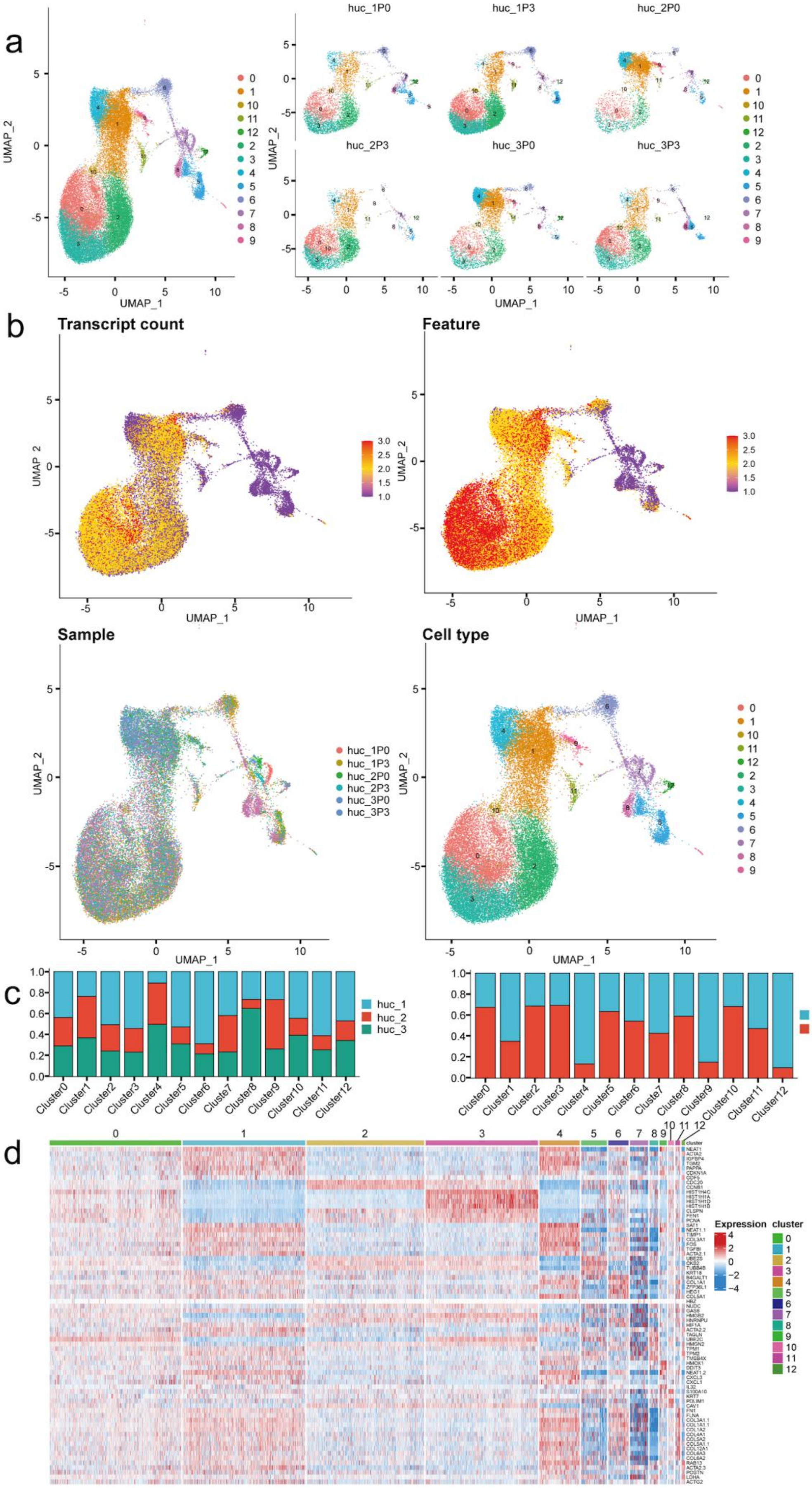

Figure S4. Primary data analysis of 13 clusters. a) The distribution of clusters from the six

samples in UMAP. **b**) UMAP visualization based on transcript count, transcript features, sample source and cell type. **c**) The proportion of each cluster in different donors and passages. **d**) Heat map shows the top DEGs of each cluster.

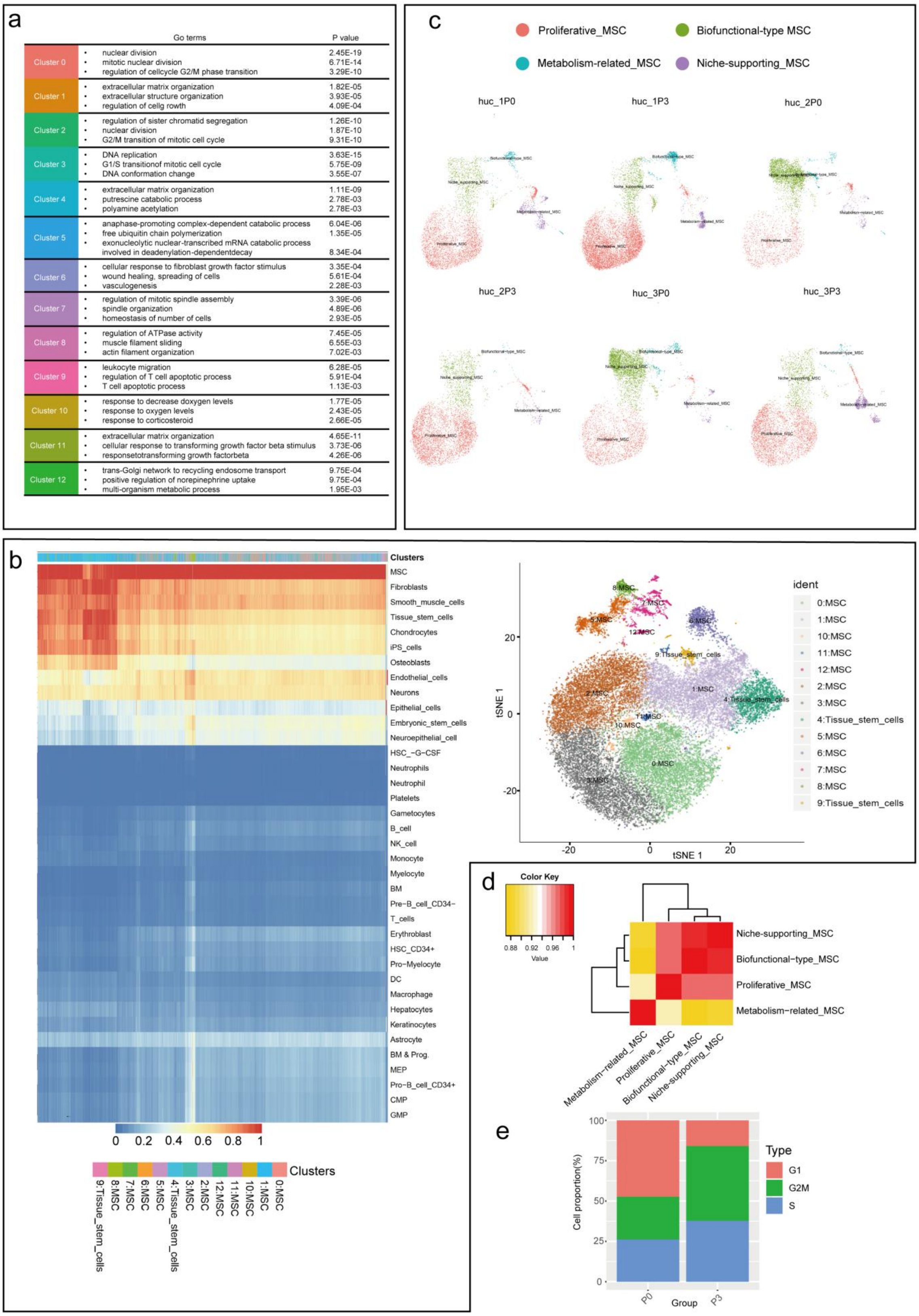

Figure S5. Cell-type annotation. a) The GO enrichment analyses of 13 clusters. **b)** Cell-type identification analysis using SingleR. **c)** The distribution of four WJ-MSCs subpopulations (proliferative_MSC, niche-supporting_MSC, metabolism-related_MSC and biofunctional-

type_MSC) from the six samples. **d**) The pairwise subpopulation correlation analysis. **e**) Cell cycle distribution of P0 and P3 cells.

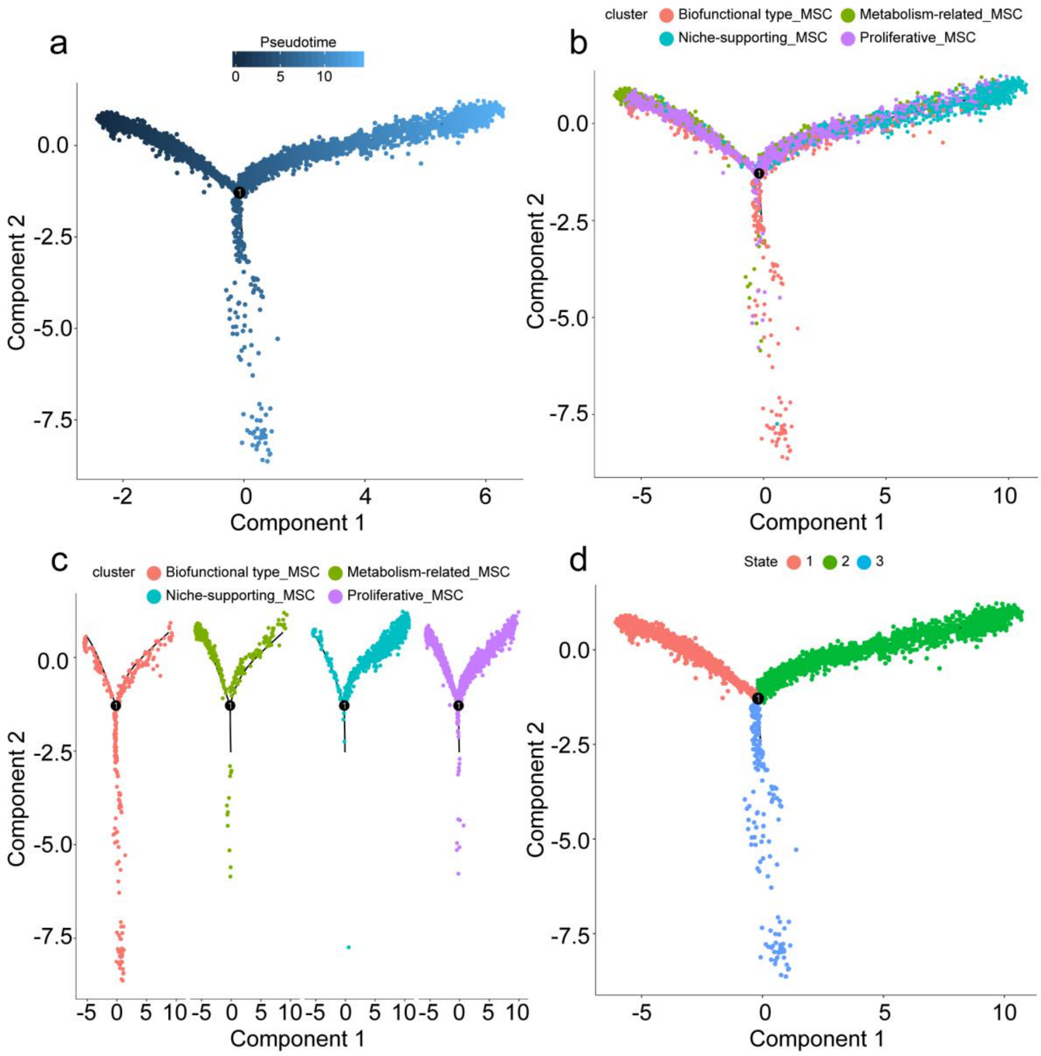

Figure S6. Transition state analysis of four WJ-MSC subpopulations in P0 sample. a) Pseudotemporal ordering trajectory map (proliferative_MSC, biofunctional-type_MSCs, niche-supporting_MSCs and metabolism-related_MSCs). The colours from dark to light represent the order of pseudo-time. **b-c)** Pseudotemporal ordering of proliferative_MSC, biofunctional-type_MSCs, niche-supporting_MSCs and metabolism-related_MSCs. **d)** Monocle analysis of WJ-MSC subpopulations trajectories-state.

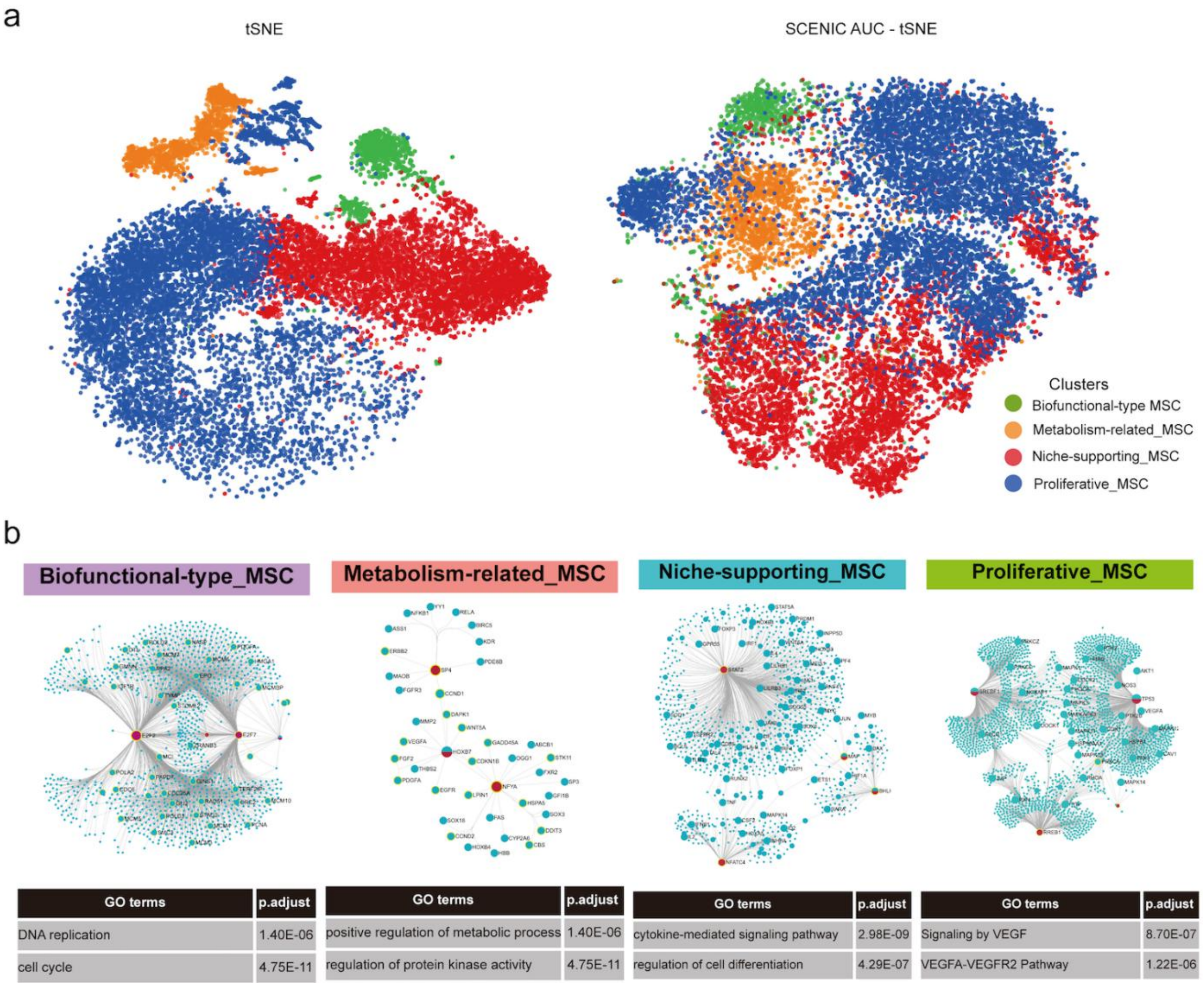

Figure S7. Transcription factor analysis among WJ-MSC subpopulations. a) SCENIC data dimensionality reduction. **b)** The miRNet database was used to predict the target genes and functional enrichment of the top 5 TFs in each cell subpopulation. Red nodes represent TFs, and light blue nodes represent predicted target genes.

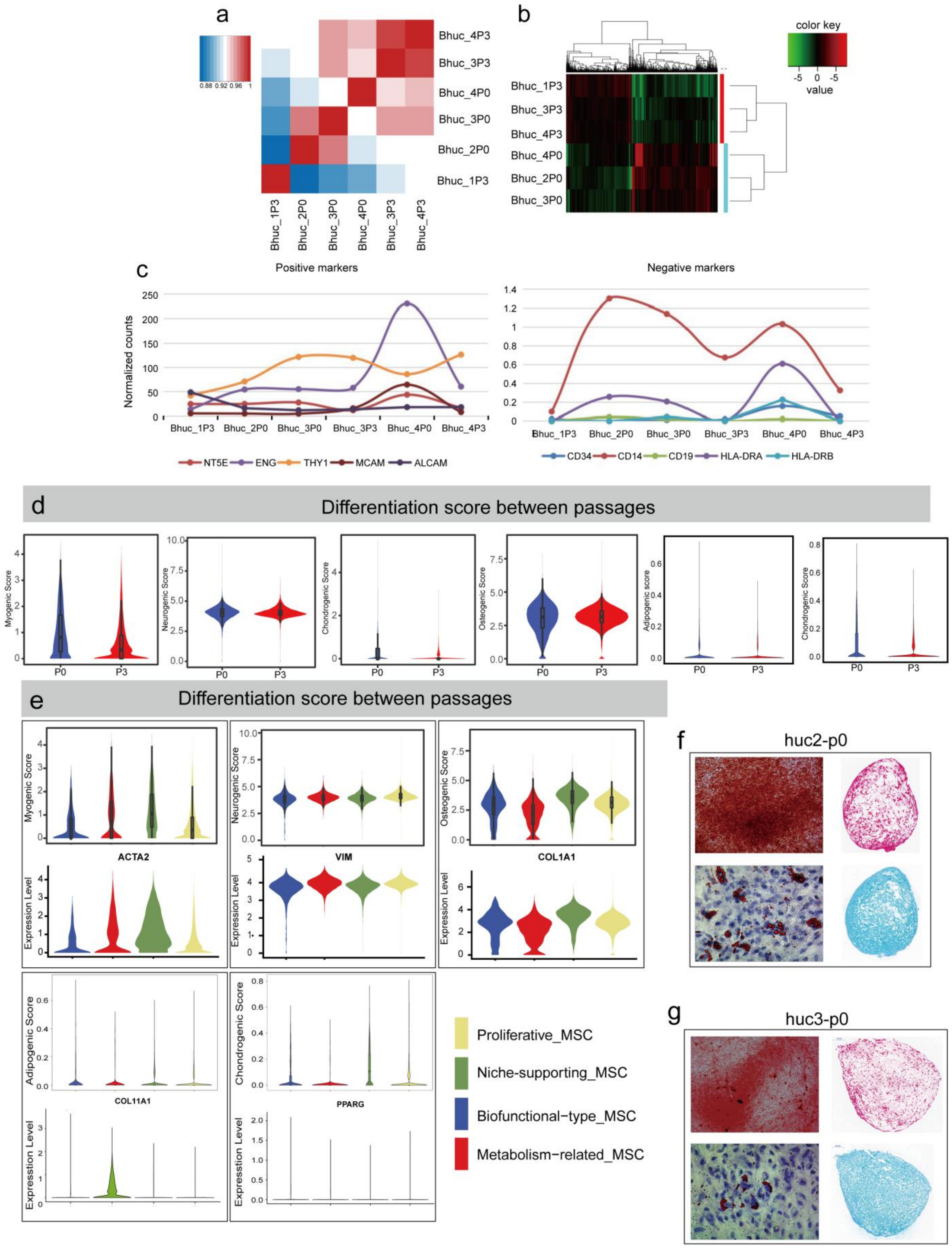

Figure S8. Transcriptional differences between P3 and P0 WJ-MSCs in Bulk-level and prediction of differentiation potential of different subpopulations. a) Correlation analysis between P3 and P0 WJ-MSCs samples. **b)** The clustering heatmap of P3 and P0 WJ-MSCs

DEGs pattern. **c)** The expression of surface markers in different passages of WJ-MSCs was evaluated from Bulk level. High expression of MSCs surface markers NT5E, ENG, THY1, MCAM and ALCAM, low expression of hematopoietic cell surface markers CD34, CD14, CD19, HLA-DRA and HLA-DRB. **d)** Differentiation score between passages. **e)** Differentiation score among four WJ-MSCs subpopulations. **f-g)** Differentiation assay for WJ-MSCs in different donors.

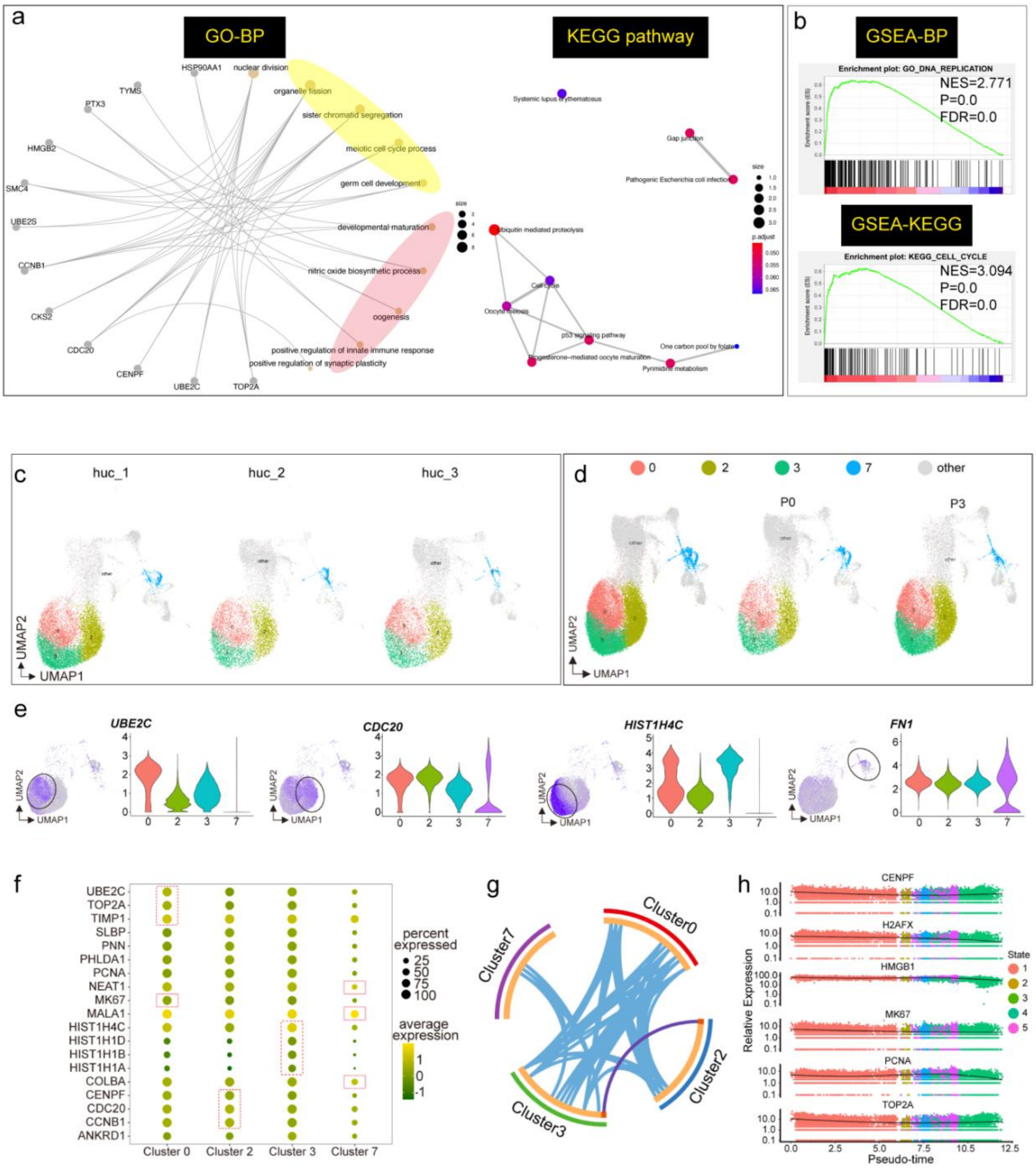

Figure S9. Subpopulation analysis of proliferative_MSCs. a) Representative Gene

Ontology biological process (GO-BP) terms and KEGG pathways showing enriched expression in proliferative_MSCs. **b)** Gene set enrichment analysis (GSEA) plots showing pathways enriched in proliferative_MSCs. **c-d)** The UMAP plot and overview of the four proliferative_MSC subsets (clusters 0, 1, 2, 3, and 7). **e)** UMAP and violin visualization of marker genes of clusters 0, 1, 2, 3, and 7. Color represents subset. **f)** Dot plot shows marker genes of clusters 0, 2, 3, and 7. **g)** Loop diagram indicating that the genes of clusters 0, 2, 3 and 7 have shared functions. **h**) Differential gene expression along pseudotime trajectory.

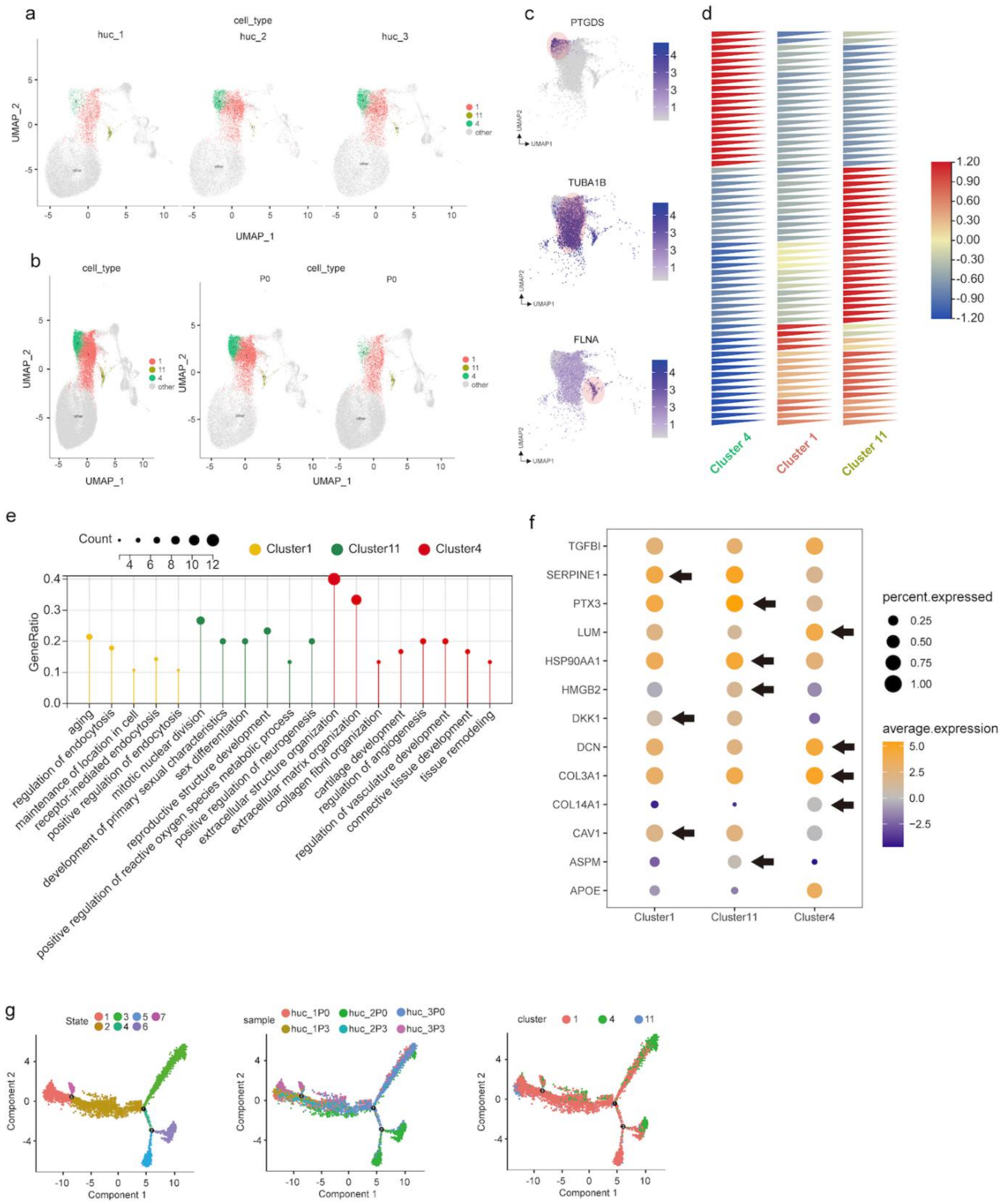

Figure S10. Subpopulation analysis of niche-supporting_MSCs. a-b) The UMAP plot and overview of the niche-supporting_MSCs subsets (clusters 1, 4, and 11). **c**) UMAP visualization of marker genes of cluster1, 4, and 11. **d**) Heatmaps showing the top 20 marker genes of clusters 1, 4, and 11. **e)** GO-BP showing the biological functions enriched in clusters 1, 4, and 11. Color represents the cell subsets. **f)** Dot plot showing the characteristic genes of clusters 1, 4, and 11. **g**) Cell ordering from different states, samples and clusters along the

pseudotime trajectory.

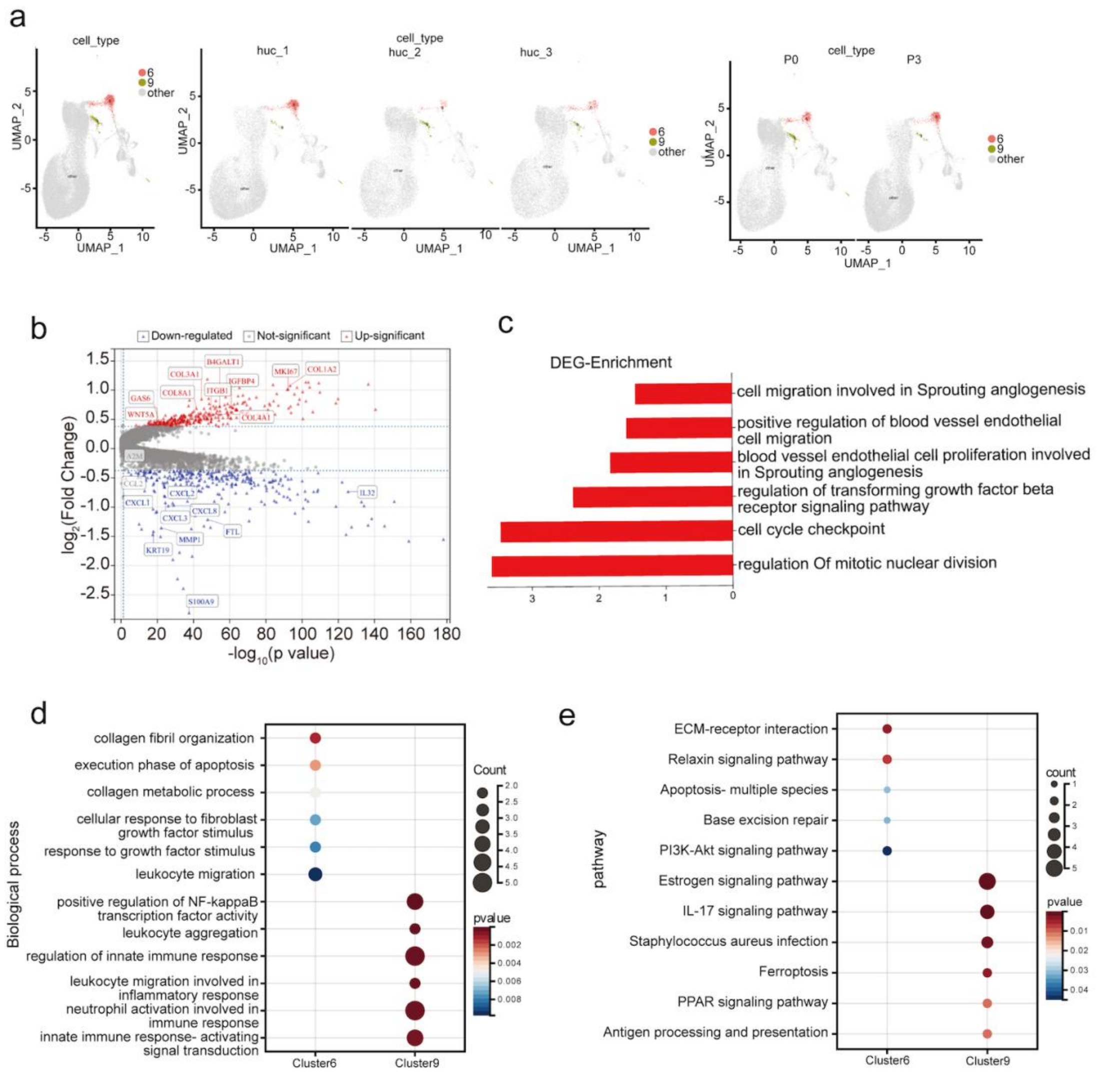

Figure S11. Subpopulation analysis of biofunctional type_MSCs. a) The UMAP plot and overview of the biofunctional type_MSCs (clusters 6 and 9). **b)** Volcano maps showing DEGs in cluster 6 and cluster 9. A fold change > 1.3 and $p < 0.05$ were as the thresholds set for upregulated genes. Specific genes are marked in the figure. **c)** DEGs enrichment analysis of P3 and P0 biofunctional type_MSCs. **d**) GO-BP analysis of cluster 6 and 9. **e**) KEGG pathway analysis of cluster 6 and 9.

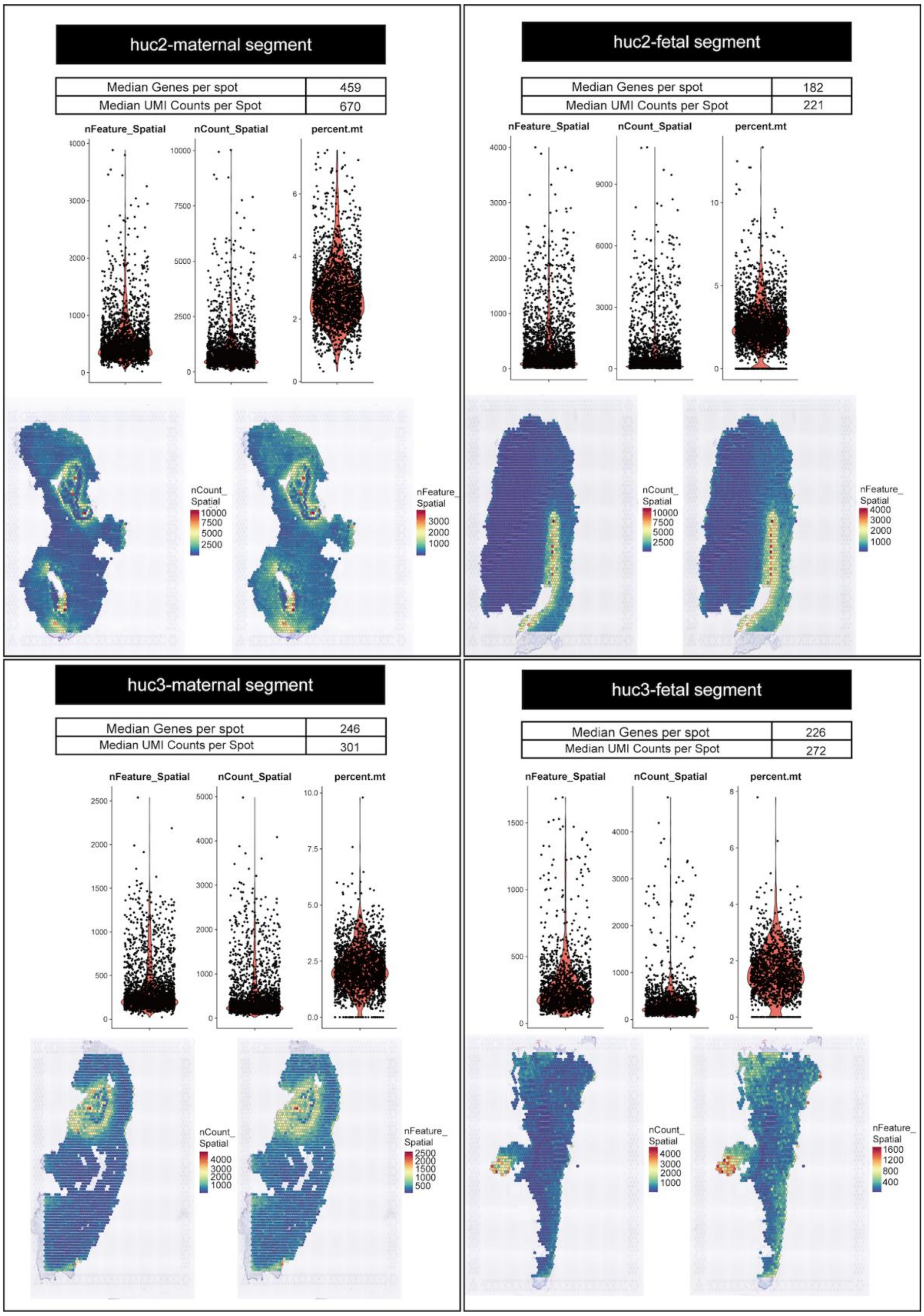

Figure S12. ST data quality assessment.

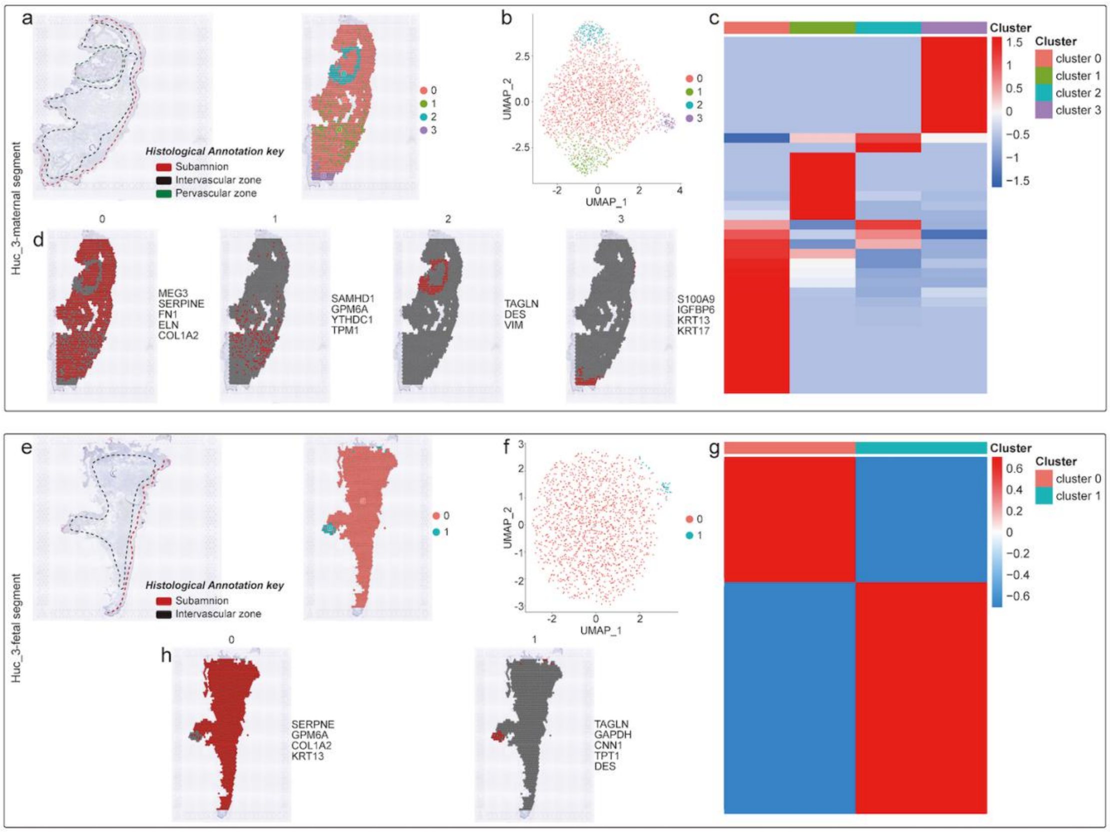

Figure S13. ST analysis of huc_3 UC tissue. a) Spatial distribution and definitions of cell types in the huc_3 maternal segment. **b**) UMAP projection of cells huc_3 maternal segment. **c**) Gene expression heatmap of the huc_3 maternal segment. **d**) Spatial expression pattern of cell types in the huc_3 maternal segment. **e)** Spatial distribution and definitions of cell types in the huc_3 fetal segment. **e**) UMAP projection of cells huc_3 fetal segment. **f)** Gene expression heatmap of the huc_3 fetal segment. **g)** Gene expression heatmap of the huc_3 fetal segment. **h**) Spatial expression pattern of cell types in the huc_3 fetal segment.

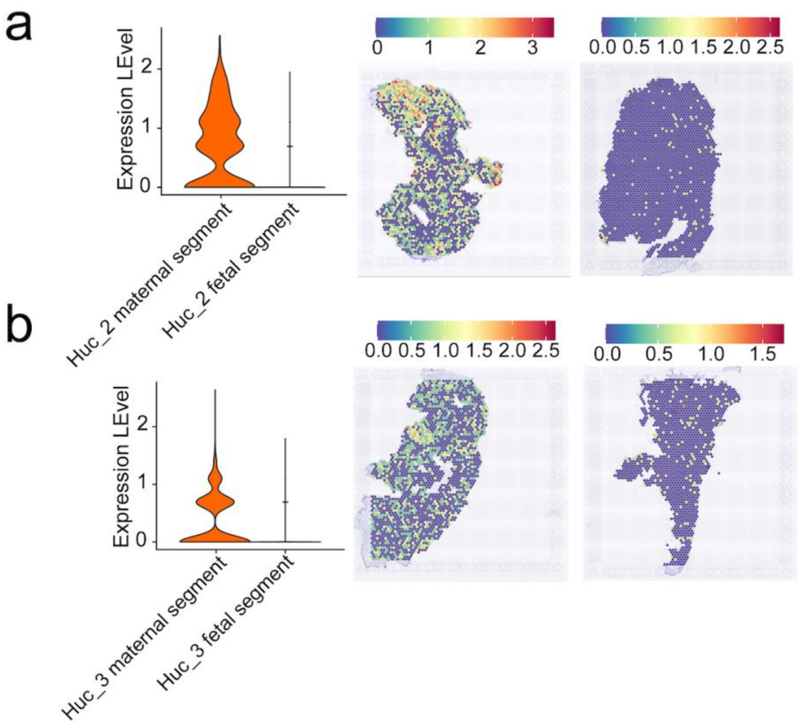

Figure S14. DLK1 gene expression levels in different regions of WJ tissue. a) Expression of DLK1 in maternal segment and fetal segment of huc_2 sample. **b)** Expression of DLK1 in maternal segment and fetal segment of huc_3 sample.

Table S1. Clinical characteristics of umbilical cord donors.

Donor ID	Age	Gestati-onal week	Delivery mode	Neonatal weight (Kg)	Neonatal sex	Number of pregnancy	History of genetic diseases	Length of umbilical cord(cm)	WBC (10^9/L)	Lympho-cyte (10^9/L)
Huc-1	31	39+2	caesarean section	4.18	Male	1	N	40	7.40	19.80
Huc-2	35	38+4	caesarean section	3.20	Female	2	N	38	14.62	14.60
Huc-3	41	37+5	caesarean section	3.05	Female	2	N	40	9.56	20.70

Donor ID	Monocyte (10^9/L)	Blood glucose (mmol/L)	Hemoglobin (g/L)	ALT (U/L)	AST (U/L)	Total Protein (g/L)	BUN (mmol/L)	Cre (µmoI/L)	Meconium-stained	APTT (s)
Huc-1	6.60	4.37	138	12	13	64.3	2.8	40.0	N	34.0
Huc-2	6.80	4.16	123	13	16	62.0	4.1	40.9	N	33.9
Huc-3	6.60	4.07	117	13	15	64.1	3.0	42.2	N	31.3
