# Supplementary material for: Single‐Cell and Spatial Transcriptomics Decodes Wharton's Jelly‐Derived Mesenchymal Stem Cells Heterogeneity and a Subpopulation with Wound Repair Signatures
Source: Adv Sci (Weinh). 2022 Dec 11;10(4):2204786. doi: 10.1002/advs.202204786 (PMC9896049; doi:10.1002/advs.202204786)
Supplement: Supplementary file 2 — Supporting Table 1 [file ADVS-10-2204786-s001.pdf]

Table S1. Clinical characteristics of umbilical cord donors.

Donor ID	Age	Gestati-onal week	Delivery mode	Neonatal weight (Kg)	Neonatal sex	Number of pregnancy	History of genetic diseases	Length of umbilical cord(cm)	WBC (10^9/L)	Lympho-cyte (10^9/L)
Huc-1	31	39+2	caesarean section	4.18	Male	1	N	40	7.40	19.80
Huc-2	35	38+4	caesarean section	3.20	Female	2	N	38	14.62	14.60
Huc-3	41	37+5	caesarean section	3.05	Female	2	N	40	9.56	20.70

Donor ID	Monocyte (10^9/L)	Blood glucose (mmol/L)	Hemoglobin (g/L)	ALT (U/L)	AST (U/L)	Total Protein (g/L)	BUN (mmol/L)	Cre (μmol/L)	Meconium-stained	APTT (s)
Huc-1	6.60	4.37	138	12	13	64.3	2.8	40.0	N	34.0
Huc-2	6.80	4.16	123	13	16	62.0	4.1	40.9	N	33.9
Huc-3	6.60	4.07	117	13	15	64.1	3.0	42.2	N	31.3
